# PAK1 inhibitor NVS‐PAK1‐1 preserves dendritic spines in amyloid/tau exposed neurons and 5xFAD mice

**DOI:** 10.1002/alz.71033

**Published:** 2025-12-26

**Authors:** Tao Yang, Hasi Huhe, Sean‐Paul Williams, Sukhneet Kaur, Yeonglong Albert Ay, Zachary W. Davis‐Gilbert, Gregory A. Cary, Carolyn Paisie, Robert R. Butler, Jesse Wiley, Ranjita Betarbet, Haian Fu, Duc Duong, Nicholas T. Seyfried, Karina Leal, Gregory W. Carter, Aled Edwards, Allan I. Levey, Jacob L. Capener, David H. Drewry, Mohammad A. Hossain, Hans J. Oh, Alison D. Axtman, Stacey J. Sukoff Rizzo, Frank M. Longo, The Emory‐Sage‐SGC‐Jax TREAT‐AD Center

**Affiliations:** ^1^ Department of Neurology and Neurological Sciences Stanford University School of Medicine Stanford California USA; ^2^ Department of Medicine–Aging Institute University of Pittsburgh Pittsburgh Pennsylvania USA; ^3^ Structural Genomics Consortium UNC Eshelman School of Pharmacy University of North Carolina at Chapel Hill Chapel Hill North Carolina USA; ^4^ The Jackson Laboratory Bar Harbor Maine USA; ^5^ The Emory‐Sage‐SGC‐Jax TREAT‐AD Center Emory USA; ^6^ The Jackson Laboratory for Genomic Medicine Farmington Connecticut USA; ^7^ Sage Bionetworks Seattle Washington USA; ^8^ Department of Neurology Emory University Atlanta Georgia USA; ^9^ Department of Pharmacology and Chemical Biology Emory University Atlanta Georgia USA; ^10^ Emory Integrated Proteomics Core (EIPC) Emory University Atlanta Georgia USA; ^11^ Department of Department of Biochemistry Emory University Atlanta Georgia USA; ^12^ Department of Medical Genetics and Medical Biophysics University of Toronto Toronto Ontario Canada; ^13^ Department of Neurobiology University of Pittsburgh School of Medicine Pittsburgh Pennsylvania USA; ^14^ Wu Tsai Neuroscience Institute Stanford University Stanford California USA

**Keywords:** Alzheimer's disease, NVS‐PAK1‐1, oligomeric amyloid beta, oligomeric tau, p21‐activated kinase 1, p21‐activated kinase 1 inhibitor, spine integrity

## Abstract

**INTRODUCTION:**

Synaptic spine loss in Alzheimer's disease (AD) contributes to cognitive decline. p21‐activated kinase 1 (PAK1), a regulator of spine integrity, is aberrantly activated in AD. We investigated whether PAK1 inhibition might preserve dendritic spines in vitro and in vivo.

**METHODS:**

Oligomeric amyloid beta (oAβ) or tau (oTau) were applied to hippocampal neurons ± NVS‐PAK1‐1, a selective PAK1 inhibitor. NVS‐PAK1‐1 was orally administered to 5xFAD mice. The effects of NVS‐PAK1‐1 treatment on PAK1 activity, spine density, and the proteome were assessed using phospho‐PAK1 (pPAK1) western blotting, Golgi staining, and mass spectrometry for proteomic analyses.

**RESULTS:**

NVS‐PAK1‐1 prevented oAβ and oTau–induced spine loss in vitro. In 5xFAD mice, NVS‐PAK1‐1 demonstrated brain exposure after oral administration and reduced PAK1 activation, prevented spine loss, and partially normalized synaptic proteomic signatures in females in absence of alterations in brain or plasma Aβ.

**DISCUSSION:**

PAK1 inhibition enhances spine resilience in AD models, supporting its therapeutic potential.

**Highlights:**

- p21‐activated kinase 1 (PAK1) inhibitors prevent oligomeric amyloid beta (oAβ) and oligomeric tau–induced spine loss and dendritic degeneration in cultured mouse hippocampal neurons.
- NVS‐PAK1‐1, a selective PAK1 inhibitor, protects against oAβ‐induced spine loss in a dose‐dependent manner (EC_50_ = 2 nM).
- Oral administration of NVS‐PAK1‐1 achieves brain penetration and bioavailability in normal CD‐1 mice, and target engagement in 5xFAD mice.
- Chronic NVS‐PAK1‐1 treatment mitigates spine loss in the somatosensory cortex of 6‐month‐old 5xFAD female mice.
- Chronic treatment with NVS‐PAK1‐1 restores proteomic abundance of actin cytoskeleton and dendritic spine‐associated proteins, including cofilin 2 and pyruvate dehydrogenase kinases, downstream of PAK1 in young 5xFAD female mice showing spine resilience.
- Clinical oncology trials with other PAK1 inhibitors support potential repurposing or novel compound development for Alzheimer's disease trials.

## 1 BACKGROUND

Alzheimer's disease (AD) is characterized by progressive cognitive decline associated with dendritic spine loss and dendritic degeneration. Synaptic impairment in AD is driven by toxic oligomers of amyloid beta (oAβ)^1^ and tau (oTau),^2^ which disrupt actin cytoskeletal dynamics, resulting in synaptic dysfunction and degeneration.^3^ Dendritic spines play a crucial role in cognitive resilience, as their density, morphology, and head diameter shape memory performance and remodel dynamically in response to AD pathology, genetic risk, and aging.^4^, ^5^, ^6^, ^7^ Preserving and stabilizing dendritic spines may help maintain synaptic connectivity and slow cognitive decline. The p21‐activated kinase 1 (PAK1) protein, a key regulator of actin remodeling, is crucial for maintaining neuronal structure and synaptic integrity.^8^ PAK1 functions upstream of cofilin, an actin‐binding protein critical for dendritic spine integrity through the PAK1 → GTPase → LIMK → cofilin signaling cascade. Disruption of this pathway compromises spine integrity,^9^ highlighting PAK1 as a potential therapeutic target in AD.

PAK1 expression is dynamically regulated across the human lifespan, showing involvement in both neurodevelopment and neurodegeneration.^10^ PAK1 activation through autosomal dominant gain‐of‐function mutations causes neurodevelopmental disorders with cognitive impairment.^11^ In human AD brains, aged Tg2576 transgenic mice, and in oAβ‐treated cultured hippocampal neurons, PAK1 is aberrantly activated and translocated from the cytosol to ectopic membrane locations, where it co‐localizes with the small GTPase Rac. This PAK1 translocation is associated with rapid F‐actin loss and decreased post‐synaptic density protein 95 (PSD‐95), both critical for synaptic stability.^12^ Fibrillar Aβ1‐42 (fAβ) also enhances PAK1 and cyclin‐dependent kinase 5 activity, altering the LIMK1–cofilin signaling axis and further disrupting actin homeostasis.^13^ Additionally, oAβ induces excessive phospho‐PAK1 (pPAK1) activation, driving abnormal actin remodeling characterized by elongated F‐actin extensions and tunneling nanotubes (TNT)‐like conduits between cells. Inhibition of PAK1 with an inhibitor (IPA‐3) prevents these pathological changes.^14^ Natural compounds like curcumin suppress Aβ‐induced PAK1 activity and prevent pPAK1 translocation in both cultured hippocampal neurons and aged Tg2576 mice, reducing synaptotoxicity, suggesting that PAK1 inhibition may offer neuroprotection in certain AD models.^15^

More broadly, PAK1 has been implicated in cancer, neurofibromatosis, diabetes, and hypertension, highlighting its targeting as having broad therapeutic potential.^16^ Multiomics analyses from large‐scale AD consortia including the Accelerating Medicines Partnership for Alzheimer's Disease (AMP‐AD) and the Target Enablement to Accelerate Therapy Development for AD (TREAT‐AD) have identified PAK1 as a high‐priority target, ranking in the top 3% of genome‐wide targets, with high genetic and multiomics risk scores across multiple AD biodomains.^17^, ^18^ In models of synaptopathy, PAK1 knockout (KO) in Fragile X syndrome (FXS) mice rescued synaptic and cognitive deficits—a finding further supported by pharmacological inhibition using non‐selective PAK inhibitors.^19^ However, in 3xTg‐AD mice, PAK1 KO or reduced PAK activity worsened synaptic and cognitive impairments and elevated phosphorylated tau (p‐tau) levels in aged animals, despite having no effect on Aβ pathology.^20^, ^21^ These conflicting findings demonstrate the dynamic nature of PAK1 function, dependent on disease context and progression. These contradictory findings underscore the need for central nervous system (CNS)–optimized PAK1 selective modulator compounds to investigate the complexity of PAK1‐related mechanisms.

**RESEARCH IN CONTEXT**

1. **Systematic review**: The authors reviewed published literature through PubMed and examined relevant conference abstracts and presentations. Alzheimer's disease (AD) remains without sufficiently effective treatment despite extensive research, largely due to its complex pathology involving amyloid accumulation, tau pathology, and synaptic loss. Recent multiomics studies from Accelerating Medicines Partnership for Alzheimer's Disease and Target Enablement to Accelerate Therapy Development for AD identified p21‐activated kinase 1 (PAK1) as a potential therapeutic target due to its aberrant activation in AD.
2. **Interpretation**: We demonstrate that the selective and allosteric PAK1 inhibitor NVS‐PAK1‐1 prevents dendritic spine loss induced by oligomeric amyloid beta and tau in vitro, and in 5xFAD mice, without altering amyloid level. Proteomic analyses revealed modulation of synaptic pathways associated with actin dynamics and spine maintenance, supporting a mechanism distinct from amyloid clearance.
3. **Future directions**: These findings support PAK1 as a novel target for synaptic protection in AD. Further studies will optimize NVS‐PAK1‐1 and related PAK1 inhibitors for clinical use, assess long‐term effects, and explore applications to other neurodegenerative diseases.

Given aberrant activation of PAK1 at various AD stages,^11^, ^12^ we hypothesize that selective PAK1 inhibitors might counteract pathological degrees of activation, potentially maintaining synaptic integrity. Furthermore, PAK1 inhibition would mitigate dendritic spine loss in cultured neurons’ exposure to pathological forms of Aβ or tau and in AD mice. To test this hypothesis, we evaluated the effects of NVS‐PAK1‐1, a highly selective allosteric PAK1 inhibitor, on spine density using in vitro and in vivo models as well as its impact on mouse brain proteome signatures. NVS‐PAK1‐1 exhibits exquisite kinome‐wide selectivity and no significant cross‐reactivity with a broad panel of proteases, receptors, and bromodomains.^22^ Pharmacokinetic (PK) and pharmacodynamic (PD) studies confirmed its brain penetration and target engagement. Notably, conjugate forms of NVS‐PAK1‐1 have been developed as potential cancer therapeutics.^23^ Our study aims to establish foundational evidence for pursuing PAK1 inhibition as a therapeutic strategy for AD‐related synaptic dysfunction.

## 2 MATERIALS AND METHODS

### 2.1 Materials

For in vitro study, NVS‐PAK1‐1 (Cat# 6132), a selective allosteric PAK1 and its inactive control compound NVS‐PAK1‐C (Cat#6133), and other PAK1 inhibitors G‐5555 (Cat#6051) and AZ13705339 (Cat#6177) were purchased from Tocris. All the compounds were obtained with a purity level > 99.8%. The hexafluoroisopropanol (HFIP)‐treated Aβ_1‐42_ peptide form (Aβ42, Cat# A‐1163‐2) and human recombinant Tau‐441 (2N4R, Cat# T‐1001‐2) were purchased from rPeptide. oAβ were prepared as previously described.^24^ Briefly, the HFIP‐treated form was resuspended in anhydrous dimethyl sulfoxide (Me_2_SO; Sigma‐Aldrich) to a final concentration of 5 mM, then diluted to 80 µM in phosphate‐buffered saline (PBS) and incubated at 4°C for 16 to 24 hours. oTau were prepared as previously described;^25^ briefly, human recombinant tau was dissolved to a final concertation of 5 µM in MES buffer (4‐morpholineethanesulfonic acid hydrate, pH 6.5), dithiothreitol (DTT; 10 µM final concentration; Sigma) was then added, and the solution was incubated at 55°C for 10 minutes. Subsequently, heparin was added to a final concentration of 5 µM (Thermo Fisher Scientific, H19) to induce aggregation. The mixture was then incubated in a shaking incubator at 1000 rpm for 4 hours at 37°C. All other chemicals were purchased from Sigma‐Aldrich Corp, unless otherwise stated.

### 2.2 Hippocampal neuron cultures and treatments

For in vitro study, all animal procedures were conducted in accordance with the National Institutes of Health Guide for the Care and Use of Laboratory Animals using protocols approved by the institutional animal care and use committee at Stanford University. Timed pregnant C57BL/6 mice (Strain code: 027) were purchased from Charles River Laboratories and delivered on embryonic day 15 (E15), after which E16 embryos were used for primary hippocampal cultures. Primary embryonic mouse hippocampal neuron cultures were plated on poly‐D‐lysine‐coated (10 µg/mL) glass cover slips at 40,000 to 50,000 cells per well in 12‐well plates (Corning Life Sciences). Cells were seeded in plating medium DMEM/F12 supplemented with 10% fetal bovine serum and 1 × penicillin/streptomycin (PS) for 2 hours. Medium was then changed to B‐27 Plus Neuronal Culture System (Invitrogen) supplemented with PS, and 2 mM glutamine. After 14 days, neurons demonstrate a mature neuron phenotype with respect to neuronal dendritic spines and functional synapses. At 14 days, cells were treated for 24 hours with either oAβ (1 µM) or oTau (50 nM), with or without PAK1 inhibitors added concomitantly (NVS‐PAK1‐C, 10 nM; NVS‐PAK1‐1, 10 nM; AZ13705339, 1 nM; G5555, 5 nM).

### 2.3 Immunofluorescence

Immunofluorescence was performed as described previously.^24^ Briefly, cultured hippocampal neurons were fixed in 4% formaldehyde for 20 minutes, permeabilized for 6 minutes in 80% ice‐cold methanol, and incubated with primary antibodies at 4°C overnight. Primary antibodies used were rabbit polyclonal MAP2 antibody for labeling dendrites (1:1000, #8707, Cell Signaling) and mouse monoclonal drebrin antibody for labeling dendritic spines (1:300; Cat#ADI‐NBA‐110, Enzo). Secondary antibodies consisted of donkey antirabbit or antimouse conjugated with either FITC or Cy3 (1:400; Jackson ImmunoResearch Laboratories, Inc.).

### 2.4 Synthesis of NVS‐PAK1‐1

For in vivo studies, custom bulk synthesis of the PAK1 selective allosteric inhibitor NVS‐PAK1‐1 (CAS#1783816‐74‐9) was completed in the laboratory of Dr. Alison Axtman at University of North Carolina.

#### 2.4.1 Chemistry: general information

All reagents and solvents were purchased from verified commercial suppliers and used without further purification. Purity of 2‐amino‐5‐chlorobenzoic acid was checked for the presence of 2‐amino‐5‐bromobenzoic acid impurities (observed by nuclear magnetic resonance [NMR] and high‐performance liquid chromatography [HPLC]). Purification via column chromatography was performed using preloaded silica gel cartridges on a Biotage automated purification system. The following abbreviations are used in schemes and/or experimental procedures: equiv. (equivalent[s]), h (hours), mmol (millimoles), mg (milligrams), min (minutes), and rt (room temperature). ^1^H NMR spectra were collected for intermediates and all final compounds to confirm their identity and assess their purity. ^1^H and ^19^F NMR spectra were obtained in DMSO‐*d*_6_, CD_3_OD, CDCl_3_, or CD_3_CN and recorded using a Bruker 400 MHz instrument. Chemical shifts are reported in parts per million (ppm) and calibrated versus the shift of the deuterated solvent used. Coupling constants (*J* values) are reported in hertz (Hz) and spin multiplicities are listed as follows: singlet (s), doublet (d), doublet of doublets/triplets/quartets (dd/dt/dq), doublet of doublet of doublets (ddd), triplet (t), triplet of doublets/triplets (td/tt), quartet (q), quartet of doublets (qd), pentent (p), and multiplet (m). Liquid chromatography mass spectrometry (LCMS) analyses were executed using an Agilent 1290 Infinity II LC System equipped with an Agilent Infinity Lab PoroShell 120 EC‐C18 column (30°C, 2.7 µm particle size, 2.1 × 50 mm), eluent 5% to 95% CH_3_CN in water with 0.2% formic acid (v/v), and flow rate of 0.5 mL/min. Synthesis of PAK1 selective allosteric inhibitor NVS‐PAK1‐1 (CAS#1783816‐74‐9) followed a modified literature procedure.^22^

#### 2.4.2 Synthesis of 5‐chloro‐2‐([4‐fluoro‐2‐nitrophenyl] amino) benzoic acid


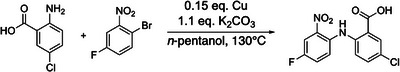


To 4 to 500 mL round‐bottom flasks (RBFs) with football stir bars attached were each added 2‐amino‐5‐chlorobenzoic acid (25 g, 1.07 equiv., 146 mmol) and 1‐bromo‐4‐fluoro‐2‐nitrobenzene (30 g, 1 Eq, 136 mmol). To each RBF was then added *n*‐pentanol (300 mL, 20.2 equiv., 3.66 mol), and the reaction mixtures were placed in a heating block on a hotplate and heated to 130°C. While warming to 130°C, the reagents fully dissolved at ≈ 100°C. At this time, K_2_CO_3_ (21 g, 1.12 equiv., 152.75 mmol) was added and heating continued for 3 min with rapid stirring. Finally, copper (powder, 10–25 µM, Sigma‐Aldrich 326453, 1.3 g, 0.15 equiv., 20.45 mmol) was added. Once the reaction temperature reached 130°C, a red precipitate slowly formed and the reaction mixture thickened, at which point stirring seized. Heating continued for 2 h to ensure reaction completion before cooling to rt. The red solid precipitates were filtered using methyl tert‐butyl ether as a transfer aid (no product is detected in the filtrate via LCMS). Solid was left on the filter with vacuum overnight (to remove any residual *n*‐pentanol). The solids were then combined in a 2 L beaker and carefully suspended in 2N HCl until bubbling seized and the suspension was acidic. The mixture was left to stir for an additional 1 h and filtered. This process was repeated once more. The crude product was then ground in a mortar and pestle and dissolved in excess (2 L) acetone and filtered to remove residual Cu powder. The filtrate was collected and dried in vacuo to give 5‐chloro‐2‐([4‐fluoro‐2‐nitrophenyl] amino) benzoic acid (combined 108.0 g, 545 mmol, 64% yield) as a red‐orange solid.

**^1^H NMR** (400 MHz, DMSO‐*d_6_*; δ, ppm) 13.78 (s, 1H), 10.88 (s, 1H), 8.01 (dd, *J* = 8.8, 3.0 Hz, 1H), 7.91 (d, *J* = 2.6 Hz, 1H), 7.70 (dd, *J* = 9.3, 4.9 Hz, 1H), 7.64–7.51 (m, 2H), 7.44 (d, *J* = 9.0 Hz, 1H).

#### 2.4.3 Synthesis of methyl 5‐chloro‐2‐([4‐fluoro‐2‐nitrophenyl]amino)benzoate


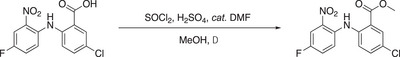


In a 1000 mL RBF with stir bar attached was added 5‐chloro‐2‐([4‐fluoro‐2‐nitrophenyl]amino)benzoic acid (73 g, 1 equiv., 0.23 mol) and MeOH (0.58 kg, 0.73 L, 77 equiv., 18 mol). Thionyl chloride (84 g, 51 mL, 3 equiv., 0.70 mol, *caution: exothermic*) was added dropwise, followed by the slow dropwise addition of H_2_SO_4_ (2.3 g, 1.3 mL, 0.1 Eq, 23 mmol, *caution: exothermic*). Finally, the addition of dimethylformamide (DMF; 0.86 g, 0.91 mL, 0.05 equiv., 12 mmol) resulted in the formation of an orange suspension. The reaction mixture was then heated to reflux for 16 h. LCMS showed only partial conversion. Additional thionyl chloride (84 g, 51 mL, 3 equiv., 0.70 mol, *caution: exothermic*) was added and the reaction refluxed for an additional 16 h. Once complete, the reaction mixture was cooled to rt, placed in an ice bath, diluted with water, and filtered. The solid was then transferred to a 2 L beaker, suspended in sat. sodium bicarbonate (1.5L), and stirred for 2 h. The solid precipitate was filtered and rinsed with 3 × 200 mL sat. sodium bicarbonate and then washed profusely with water until washings were light‐orange/colorless (≈ 1–2L). The solid was dried overnight on a Buchner funnel, transferred to a 1000 mL RBF, dried on a rotovap (30 mmbar, 60°C) for 2 h, and further dried on a Schlenk line to give methyl 5‐chloro‐2‐([4‐fluoro‐2‐nitrophenyl]amino)benzoate (72.5 g, 0.23 mol, 95%) as an orange solid.

**^1^H NMR** (400 MHz, DMSO‐*d_6_*; δ, ppm) 10.65 (s, 1H), 8.02 (dd, *J* = 8.9, 3.0 Hz, 1H), 7.92 (d, *J* = 2.6 Hz, 1H), 7.67 (dd, *J* = 9.3, 4.9 Hz, 1H), 7.59 (ddt, *J* = 9.3, 6.6, 3.4 Hz, 2H), 7.47 (d, *J* = 8.9 Hz, 1H), 3.89 (s, 3H).

#### 2.4.4 Synthesis of methyl 5‐chloro‐2‐([2,2‐difluoroethyl][4‐fluoro‐2‐nitrophenyl]amino)benzoate


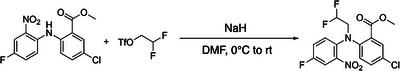


To 4 to 500 mL RBFs with stir bars attached were each added methyl 5‐chloro‐2‐([4‐fluoro‐2‐nitrophenyl]amino)benzoate (25 g, 1.0 Eq, 77 mmol) and DMF (45 mL, 45 equiv.). The reaction mixtures were placed in an ice bath and stirred at 0°C for 30 min. To each RBF was then added NaH (4.9 g, 1.6 equiv., 123.2 mmol, *caution: exothermic and gas formation)* portion‐wise while maintaining an internal temperature of ≈ 0°C. After complete addition, stirring continued for an additional 1 h. At 0°C, 2,2‐difluoroethyl trifluoromethanesulfonate (27.7 g, 16 mL, 1.5 equiv., 120 mmol) was added dropwise over 1 h to the reaction mixture. The reaction mixture was then allowed to warm to rt and continue stirring at rt until completion as monitored via LCMS. The reaction mixture was then condensed to reduce the content of DMF, diluted with 1N HCl (150 mL), and extracted with EtOAc (150 mL, 3X). The organic fractions were washed with saturated NaHCO_3_ and brine. The organics were then dried over anhydrous Na_2_SO_4_, filtered, and dried in vacuo. The crude mixture was purified by flash column chromatography using EtOAc:Hexanes (0 to 50% over 20 min). The product fractions were collected and dried to give methyl 5‐chloro‐2‐([2,2‐difluoroethyl][4‐fluoro‐2‐nitrophenyl]amino)benzoate (88 g, 226 mmol, yield 74%) as a yellow solid.

**^1^H NMR** (400 MHz, CDCl_3_; δ, ppm) 7.59 (d, *J* = 2.6 Hz, 1H), 7.44–7.35 (m, 3H), 7.32–7.25 (m, 1H), 7.18 (d, *J* = 8.7 Hz, 1H), 6.13 (tt, *J* = 55.5, 4.1 Hz, 1H), 4.14 (td, *J* = 13.4, 4.2 Hz, 2H), 3.62 (s, 3H).

#### 2.4.5 Synthesis of 2‐chloro‐5‐(2,2‐difluoroethyl)‐8‐fluoro‐5,10‐dihydro‐11H‐dibenzo[*b*,*e*][1,4]diazepin‐11‐one


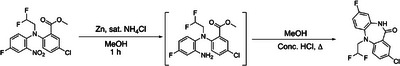


To a 1000 mL RBF with a large stir bar attached was added methyl 5‐chloro‐2‐([2,2‐difluoroethyl][4‐fluoro‐2‐nitrophenyl]amino)benzoate (37.6 g, 1 equiv., 96.8 mmol) and MeOH (620 g, 783 mL, 200 equiv., 19.4 mol). The reaction mixture was warmed to 40°C and Zn dust (44.3 g, 7 equiv., 677 mmol) was added. After 15 min, the mixture cooled to 0°C and sat. ammonium chloride (51.8 g, 138 mL, 7 M, 10 equiv., 968 mmol) was added dropwise. After complete addition and stirring for 30 min, additional Zn dust (22.2 g, 3.5 equiv., 339 mmol) was added followed by dropwise addition of additional sat. ammonium chloride (25.9 g, 69.1 mmol, 5 equiv.). The final reaction mixture was light yellow with a gray suspension, indicating reaction completion. The reaction mixture was filtered to remove Zn, and the solids were washed with excess MeOH. MeOH was removed in vacuo and the solid gum was partitioned between EtOAc and deionized H_2_O. The organic layer was washed 3X with deionized H_2_O. The combined waters were extracted with EtOAc, the organics were combined, dried over MgSO_4_, filtered, and dried in vacuo. The crude material was taken directly to the next step.

To a 1000 mL RBF with a large football stir bar attached was added methyl 2‐([2‐amino‐4‐fluorophenyl][2,2‐difluoroethyl]amino)‐5‐chlorobenzoate (34.7 g, 1 Eq, 96.8 mmol) and MeOH (4 g, 5 mL, 1 Eq, 0.1 mol). To this was added con. HCl (275 g, 629 mL, 78 equiv., 7.5 mol). A reflux condenser was then attached, and the reaction mixture was refluxed for 2 days. The reaction mixture was cooled to rt and methanol was removed in vacuo. Yellow solid precipitate was then filtered and washed profusely with sat. sodium bicarbonate and then water. Finally, the product was transferred to a 1000 mL RBF, diluted in methanol, and brought to dryness in vacuo. This process was repeated twice to give 2‐chloro‐5‐(2,2‐difluoroethyl)‐8‐fluoro‐5,10‐dihydro‐11*H*‐dibenzo[*b*,*e*][1,4]diazepin‐11‐one (29 g, 89 mmol, 92% yield over 2 steps) as a yellow solid.

**^1^H NMR** (400 MHz, DMSO‐*d_6_*; δ, ppm) 10.52 (s, 1H), 7.62–7.54 (m, 1H), 7.44–7.35 (m, 1H), 6.99 (ddd, *J* = 8.9, 8.1, 3.0 Hz, 1H), 6.89 (dd, *J* = 9.7, 3.0 Hz, 1H), 6.05 (tt, *J* = 55.2, 4.0 Hz, 1H), 4.26 (dtd, *J* = 33.8, 14.5, 4.0 Hz, 2H).

#### 2.4.6 Synthesis of *tert*‐butyl (*S*)‐3‐((2‐chloro‐5‐(2,2‐difluoroethyl)‐8‐fluoro‐5*H* dibenzo[*b*,*e*][1,4]diazepin‐11‐yl)amino)pyrrolidine‐1‐carboxylate


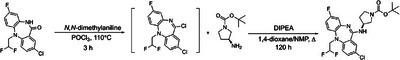


To a 500 mL RBF with a large stir bar attached was added 2‐chloro‐5‐(2,2‐difluoroethyl)‐8‐fluoro‐5,10‐dihydro‐11*H*‐dibenzo[*b*,*e*][1,4]diazepin‐11‐one (28.5 g, 1 equiv., 87.2 mmol), phosphoryl trichloride (201 g, 122 mL, 15 equiv., 1.31 mol) and *N*,*N*‐dimethylaniline (7.40 g, 7.74 mL, 0.7 equiv., 61.1 mmol). The reaction mixture was heated to 110°C for 3 h. After completion (as checked by LCMS), the reaction mixture was cooled to rt, dried in vacuo, reconstituted with toluene, and further dried. This process was repeated twice more to remove residual POCl_3_. The crude material was then taken to the next step without further purification.

To a 500 mL RBF was added 2,11‐dichloro‐5‐(2,2‐difluoroethyl)‐8‐fluoro‐5*H*‐dibenzo[*b*,*e*][1,4]diazepine (30.1 g, 1 equiv., 87.2 mmol), 1,4‐dioxane (307 g, 298 mL, 40 equiv., 3.5 mol), N‐methyl‐2‐pyrrolidone (NMP; 28.1 g, 27.3 mL, 3.25 equiv., 283 mmol), (*S*)‐3‐Aminopyrrolidine‐1‐carboxylic acid *tert*‐butyl ester (48.7 g, 47.8 mL, 3 equiv., 262 mmol), and *N,N*‐diisopropylethylamine (DIPEA; 33.8 g, 45.6 mL, 3 equiv., 262 mmol). This was heated for 3 days at 90°C. Reaction was incomplete at this time and the temperature was raised to 115°C for an additional 2 days to give complete conversion. The reaction mixture was cooled to rt and the 1,4‐dioxane was stripped. The crude was poured into 500 mL EtOAc and washed 4 × 200 mL water. Organics were then dried over MgSO_4_ and filtered. Isolute Si was added to give a suspension and dried in vacuo. The solid loaded material was then purified by flash chromatography using ethyl acetate/hexanes, and product fractions were collected and dried to give *tert*‐butyl (*S*)‐3‐((2‐chloro‐5‐(2,2‐difluoroethyl)‐8‐fluoro‐5*H*‐dibenzo[*b*,*e*][1,4]diazepin‐11‐yl)amino)pyrrolidine‐1‐carboxylate (30.5 g, 87.2 mmol, 70% yield) as a yellow solid.

**^1^H NMR** (400 MHz, DMSO‐*d_6_*; δ, ppm) 7.64–7.43 (m, 2H), 7.40–7.20 (m, 2H), 7.08 (ddd, *J* = 8.7, 5.7, 2.1 Hz, 1H), 6.73–6.57 (m, 2H), 5.94 (tdd, *J* = 55.2, 8.4, 4.3 Hz, 1H), 4.49 (s, 1H), 4.22–3.84 (m, 2H), 3.67–3.41 (m, 1H), 3.28 (s, 1H), 2.26–1.77 (m, 1H), 1.36 (d, *J* = 20.8 Hz, 9H).

#### 2.4.7 Synthesis of (*S*)‐3‐([2‐chloro‐5‐(2,2‐difluoroethyl)‐8‐fluoro‐5*H*‐dibenzo[*b*,*e*][1,4]diazepin‐11‐yl]amino)‐N‐isopropylpyrrolidine‐1‐carboxamide, bis trifluoroacetic acid, NVS‐PAK1‐1


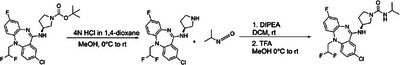


To a 1000 mL RBF with stir bar attached was added *tert*‐butyl (*S*)‐3‐([2‐chloro‐5‐(2,2‐difluoroethyl)‐8‐fluoro‐5*H*‐dibenzo[*b*,*e*][1,4]diazepin‐11‐yl]amino)pyrrolidine‐1‐carboxylate (30.5 g, 1 equiv., 61.6 mmol) and MeOH (395 g, 499 mL, 200 equiv., 12.3 mol). The mixture was cooled to 0°C. Then, 4N HCl in 1,4‐dioxane (22.5 g, 154 mL, 4 molar, 10 equiv., 616 mmol) was added dropwise and the reaction left to stir for 30 min at 0°C before warming to rt and stirring until complete by TLC. Once complete, the reaction mixture was concentrated to ≈ 50 mL. Then, DIPEA (4.62 g, 6.23 mL, 1 equiv., 35.8 mmol) was added followed by 30 g of celite to give a suspension. The reaction mixture was then concentrated to dryness in vacuo and purified by flash chromatography. The column was first flushed with 100% EtOAc to remove any residual starting materials and non‐polar impurities. The gradient was then increased to 30% MeOH (0.1% DIPEA)/EtOAc and maintained until product elution. The product fractions were collected and dried to yield (*S*)‐2‐chloro‐5‐(2,2‐difluoroethyl)‐8‐fluoro‐*N*‐(pyrrolidin‐3‐yl)‐5*H*‐dibenzo[*b*,*e*][1,4]diazepin‐11‐amine (30.5 g, 61.6 mmol, 77% yield), which contained 2 equivs of DIPEA. This material was taken directly to the next step without further purification.

**^1^H NMR** (400 MHz, MeOD; δ, ppm) 7.71–7.44 (m, 2H), 7.27 (dd, *J* = 8.9, 1.6 Hz, 1H), 7.12 (dd, *J* = 8.8, 5.5 Hz, 1H), 6.76 (dddd, *J* = 19.5, 8.3, 6.2, 3.0 Hz, 2H), 6.15–5.59 (m, 1H), 4.81–4.64 (m, 1H), 4.17–4.01 (m, 2H), 3.76 (dq, *J* = 13.3, 6.7 Hz, 4H [*DIPEA*]), 3.70–3.55 (m, 3H), 3.51–3.35 (m, 2H), 3.25 (q, *J* = 7.4 Hz, 4H, [*DIPEA*]), 2.57–2.36 (m, 1H), 2.36–2.17 (m, 1H), 1.40 (td, *J* = 7.4, 6.7, 1.8 Hz, 24H, [*DIPEA*]).

To a 500 mL RBF with stir bar attached was added (*S*)‐2‐chloro‐5‐(2,2‐difluoroethyl)‐8‐fluoro‐*N*‐(pyrrolidin‐3‐yl)‐5*H*‐dibenzo[*b*,*e*][1,4]diazepin‐11‐amine, 2 diisopropylethylamine (30 g, 1 equiv., 46 mmol), CH_2_Cl_2_ (264 g, 200 mL, 67 equiv., 3.11 mol), and DIPEA (12 g, 16 mL, 2 equiv., 93 mmol). Under vigorous stirring, 2‐isocyanatopropane (4.3 g, 5.0 mL, 1.1 equiv., 51 mmol) was added dropwise. The reaction mixture was stirred until complete by LCMS. To this mixture was added directly 50 g Isolute and the suspension was brought to dryness. An additional 100 to 200 mL CH_2_Cl_2_ was added and brought to dryness. The solid material was purified by column chromatography split across four 220 g columns. The purification process began with 0% hexanes (0.5 column volume [CV]), followed by a gradient from 0% to 100% EtOAc/hexanes (1 CV), and continued with 100% EtOAc (4–6 CV) until complete product elution. Product fractions were collected and dried to give product as a 1:1 solvate with EtOAc.

The solids were redissolved in MeOH 200 mL and brought to dryness in vacuo. This was repeated 3X to remove residual EtOAc. Once dry, the solid was again reconstituted in 200 mL MeOH and cooled in an ice bath to 0°C. To this solution was added trifluoroacetic acid (16 g, 11 mL, 3 equiv., 0.14 mol) dropwise. The mixture was stirred while warming to rt and stirring continued for 1 h before being dried in vacuo. The foamy material was reconstituted in 200 mL MeOH and dried in vacuo to give a tan–white solid foam. The solid was crushed to a fine powder and dried on a Schlenk line with gentle heating (≈ 40°C) to give (*S*)‐3‐([2‐chloro‐5‐(2,2‐difluoroethyl)‐8‐fluoro‐5H‐dibenzo[*b*,*e*][1,4]diazepin‐11‐yl]amino)‐N‐isopropylpyrrolidine‐1‐carboxamide, 2 Trifluoroacetic acid (30.2 g, 42.7 mmol) as an off‐white solid. The product was checked for purity by ^1^H NMR (DMSO‐*d_6_* and MeOD, >98%) and LCMS (> 99%; see Figures S1–S5 in supporting information).

^1^ **H NMR in DMSO‐*d_6_* matched reported literature**.^22^

**^1^H NMR** (400 MHz, DMSO‐*d_6_*; δ, ppm) 7.74 (dd, *J* = 8.8, 2.5 Hz, 1H), 7.62 (dd, *J* = 5.2, 2.5 Hz, 1H), 7.54 (d, *J* = 8.9 Hz, 1H), 7.45 (q, *J* = 7.4, 6.9 Hz, 1H), 7.14 (d, *J* = 11.9 Hz, 2H), 6.47–5.96 (m, 1H), 5.91 (d, *J* = 7.8 Hz, 1H), 4.64 (p, *J* = 5.5 Hz, 1H), 4.39 (td, *J* = 14.3, 4.1 Hz, 1H), 4.34–4.15 (m, 1H), 3.85–3.64 (m, 2H), 3.52 (ddd, *J* = 17.8, 10.2, 5.6 Hz, 1H), 3.36 (ddd, *J* = 14.9, 8.3, 3.6 Hz, 2H), 2.29 (ddt, *J* = 33.7, 13.5, 6.5 Hz, 2H), 2.03 (dq, *J* = 11.8, 5.5 Hz, 1H), 1.12–0.97 (m, 6H).

**^1^H NMR** (400 MHz, MeOD; δ, ppm) 7.95–7.63 (m, 2H), 7.56–7.44 (m, 2H), 7.24–7.14 (m, 2H), 6.01 (ttd, *J* = 55.3, 3.6, 1.7 Hz, 1H), 4.67 (dp, *J* = 6.1, 3.9, 3.3 Hz, 1H), 4.44–4.17 (m, 2H), 4.02–3.81 (m, 2H), 3.75–3.44 (m, 3H), 2.60–2.15 (m, 2H), 1.26–1.15 (m, 6H).

**^19^F NMR** (376 MHz, MeOD; δ, ppm) δ ‐77.19 (s, 6H), −117.10 (dtd, *J* = 24.9, 8.5, 5.1 Hz, 1H), −123.40 (ddt, *J* = 55.4, 17.8, 14.0 Hz, 1H), −123.78 – −124.4 (m, 1H).

**LCMS**: [M+] 480.1 *m*/*z*

### 2.5 In vivo PK studies

Initial in vivo PK studies to confirm oral bioavailability and brain exposure were conducted at Pharmaron (Beijing, China) in non‐fasted male CD‐1 mice (6–8 weeks of age; sourced from Vital River). For these studies, NVS‐PAK1‐1 was formulated in a solution of NMP solutol/PEG‐400/normal saline (v/v/v/v, 10:5:30:55) just before dosing. The dose volume was 10 mL/kg.

#### 2.5.1 Study design

First a snapshot PK compared exposure levels of oral administration (PO) versus intraperitoneal (IP) routes of administration of 10 mg/kg NVS‐PAK1‐1 (*n* = 2) to serial blood sampling at 0.5, 1, 3, and 5 hours post dose. Next, we evaluated brain and plasma PK of 10 mg/kg (1 hour pre, IP, *n* = 3). Experiments then were conducted to confirm and extend initial PK data and evaluate brain and plasma levels at doses of 1, 10, and 100 mg/kg after oral administration at 1, 3, and 8 hours post dose (*n* = 3 per dose per timepoint).

#### 2.5.2 Sample collection

Serial blood was collected via the dorsal metatarsal vein into ethylenediaminetetraacetic acid‐K2 coated tubes and centrifuged at 4°C x 4000 g for 5 minutes to obtain plasma that was aliquoted and stored at −80°C until analysis. For terminal studies, blood was collected via cardiac puncture under isoflurane anesthesia. Whole brain was homogenized at a ratio of 1:3 with PBS (W:V, 1:3).

#### 2.5.3 Sample analysis

For sample analysis liquid chromatography tandem mass spectrometry (LC‐MS/MS) was conducted using an AB Sciex Triple Quad 5500 LC/MS/MS instrument. Briefly, the desired serial concentrations of working solutions were achieved by diluting stock solution of analyte with 50% acetonitrile in water solution. Ten µL of working solutions (0.5, 1, 2, 5, 10, 50, 100, 500, and 1000 ng/mL) were added to 10 µL of the blank CD1 mouse plasma/brain homogenate to achieve calibration standards of 0.5 to 1000 ng/mL (0.5, 1, 2, 5, 10, 50, 100, 500, and 1000 ng/mL) in a total volume of 20 µL. Five quality control (QC) samples at 1 ng/mL, 2 ng/mL, 5 ng/mL, 50 ng/mL, and 800 ng/mL for plasma/brain homogenate were prepared independently of those used for the calibration curves. These QC samples were prepared on the day of analysis in the same way as calibration standards. Twenty µL standards, 20 µL QC samples, and 20 µL unknown samples (10 µL plasma/brain homogenate with 10 µL blank solution) were added to 200 µL of acetonitrile containing internal standard (IS) mixture for precipitating protein, respectively. The samples were vortexed for 30 seconds followed by centrifugation at 4°C x 2600 g for 15 minutes. The supernatant was diluted at a ratio of 1:2 with H2O (V/V, 1:2), 5 µL of diluted supernatant was injected into the LC‐MS/MS system for quantitative analysis. The lower limit of quantification (LLOQ) was 0.5 ng/mL for plasma and 1 ng/mL for brain. PK parameters were estimated by non‐compartmental model analysis using WinNonlin 8.3.

### 2.6 In vivo studies assessing target engagement, dendritic spine density, and levels of neurofilament light chain and Aβ

#### 2.6.1 Animal studies

In vivo target engagement and PK/PD studies were conducted at the University of Pittsburgh School of Medicine. Prior to study initiation, all experiments were approved by the University of Pittsburgh Institutional Animal Care and Use Committee (IACUC) with strict adherence to the Guide for the Care and Use of Laboratory Animals (NIH Publication No. 85‐23, revised 2011), and in line with Animals in Research: Reporting In Vivo Experiments (ARRIVE) guidelines.^26^ For these studies, 5xFAD hemizygous male mice on a congenic C57BL/6J background were obtained from The Jackson Laboratory (B6.Cg‐Tg(APPSwFlLon, PSEN1*M146L*L286V)6799Vas/Mmjax; JAX #034848) and bred to female C57BL/6J mice (JAX#000664) to generate litters of male and female 5xFAD and non‐transgenic wild‐type (WT) littermates that were enrolled in these studies. The 5xFAD model was specifically selected for these studies as they develop robust amyloid pathology by 6 months of age and can be used to expand on the in vitro studies that investigated PAK1 effects on oAβ. Mice were group housed (*n* = 2–4 per cage) with ad libitum food (Lab Diet 5P76, irradiated chow; Lab Diet) and automated water delivered via a lixit system (chlorinated at 2–3 ppm, pH 4–5) in a dedicated mouse housing room with a 12:12 light‐to‐dark cycle (lights on at 7:00 am). The housing and procedure room temperatures were maintained at 22 ± 2°C and 50 ± 10% humidity. All experiments were conducted during the light cycle. For genotyping, the crude genomic DNA was extracted from ear‐punch at weaning and confirmed at study end via tail snip. The primers used for genotype confirmations of 5xFAD and WT were as follows: forward primer: GCCATGAGGGCACTAATCAT/reverse primer: AATAGAGAACGGCAGGAGCA and forward primer: CTAGGCCACAGAATTGAAAGATCT/reverse primer: GTAGGTGGAAATTCTAGCATCATCC.

#### 2.6.2 Study design

For the initial multidose pilot PK/PD study in female and male 5xFAD mice (*n* = 3 per sex per treatment group), NVS‐PAK1‐1 (10 or 50 or 100 mg/kg) or vehicle control was orally administered (PO) as a single dose (Table S1 in supporting information). Serial blood was collected from *n* = 3 per treatment group at 0.5, 1, 2, and 4 hours post dose; with terminal blood and brain collected at 4 hours post dose. A staggered cohort of *n* = 3 per treatment group were assigned to serial blood collections at 0.5, 1, 2, and 8 hours post dose with terminal blood and brain collected at 4 and 24 hours post dose. Informed by exposure data from the initial PK/PD study, a subsequent chronic PK/PD study was designed to evaluate twice daily treatment of 100 mg/kg (PO) in female and male 5xFAD mice compared to age‐ and sex‐matched vehicle‐treated 5xFAD and vehicle‐treated WT littermate controls (Table S2 in supporting information). Terminal blood and brain was collected at 4 hours after the final dose. The goal of these studies was to evaluate effects of NVS‐PAK1‐1 treatment in mice with mild amyloid deposition in young 5xFAD mice at 2 to 3 months of age compared to the effects of NVS‐PAK1‐1 treatment on significant amyloid deposition in 8‐ to 9‐month aged 5xFAD mice (*n* = 9–14 per sex per treatment group at study enrollment). All in vivo target engagement and PK/PD studies were conducted under blinded conditions for age, genotype, and treatment, and the blind was maintained until after analyses were conducted. All subjects that completed the treatment regimen were included in all analyses except for mice that were euthanized prior to study completion due to welfare concerns as described below, or if confirmatory genotyping, conducted prior to unblinding, indicated incorrect genotype.

#### 2.6.3 NVS‐PAK1‐1 treatment

NVS‐PAK1‐1 was sourced from bulk, custom synthesis as described above. For in vivo target engagement and PK/PD studies in 5xFAD mice, NVS‐PAK1‐1 was formulated fresh daily in a solution of 0.25% Tween 80 (Fisher Scientific #BP338‐500), 1% hydroxypropyl methylcellulose (Millipore Sigma #09963‐500 g) in molecular grade water which was maintained as a stock solution and stored at 4°C. Drug concentrations were prepared immediately before dosing each day. The dose volume was delivered at 10 mL/kg based on each subject's daily weight collected just prior to the morning dose and administered via PO. For the chronic PD study, twice daily dosing (BID) was conducted between 7:00 and 9:00 am each morning and 3:00 to 5:00 pm each evening with the same day's formulation stored at 4°C between dosing intervals.

#### 2.6.4 Blood and tissue collection

For serial blood sampling, 50 µL of blood was collected via the tail tip route into heparin‐coated glass capillary tubes (Drummond scientific # 1‐000‐7500‐C/5) in non‐anesthetized mice. For all terminal timepoints, tissue collection was conducted at 4 or 24 hours post dose for acute studies (Tables S1–S2) and 4 hours after the final dose administrated for chronic studies. Mice were anesthetized with isoflurane to the surgical plane of anesthesia. After decapitation, trunk blood was collected into heparin‐coated tubes for terminal PK analysis with the remaining trunk blood decanted into EDTA‐coated tubes for PD analysis. Blood was kept on ice until centrifugation (typically 20–60 minutes post collection), then centrifuged at 19745 g for 10 minutes at 4°C for PK samples and 20 minutes for PD samples (e.g., plasma Aβ40, Aβ42, neurofilament light chain [NfL]), followed by storage at −80°C until analysis. The brain was quickly removed from the skull, briefly rinsed in cold PBS, and divided sagittally at the midline into left and right hemispheres. The left hemisphere without cerebellum was quickly chopped and mixed into three equally aliquoted mixed parts then placed into three cold cryotubes. Cryotubes were immediately snap frozen on dry ice and stored at −80°C until analysis. The right hemisphere was maintained intact and immediately placed on dry ice and stored at −80°C until shipped to Stanford University for Golgi staining and morphological analyses.

#### 2.6.5 PK analysis

Brain and plasma samples from 5xFAD mice were shipped to Touchstone Biosciences (Plymouth Meeting, PA, USA) for analysis of NVS‐PAK1‐1 concentrations. For plasma samples, three volumes of acetonitrile containing internal standard was added to one volume of plasma, and centrifuged at 3000 g for 10 minute; then, the supernatant was removed and analyzed by LC‐MS/MS. Calibration standards and quality controls were prepared from a 1 mg/mL stock solution and subsequently a series of working solutions in methanol:water (1:1, v/v) that are spiked into blank plasma to yield a series of calibration standard samples in the range of 1 ng/mL to 10 µg/mL and QC samples at three concentration levels (low, middle, and high). All plasma samples were treated identically to the calibration standards and QC samples. For analysis of brain, one third of the left mixed‐chopped hemisphere was homogenized in three volumes of PBS buffer (pH 7.4; v/w = 3/1). Subsequently, three volumes of acetonitrile‐containing internal standard was added to one volume of each tissue homogenate, and the mixture was vortexed, centrifuged at 3000 g for 10 minutes, and supernatants were used for LC‐MS/MS. Calibration standards were made by preparation of a 1 mg/mL stock solution and subsequently a series of working solutions in methanol:water (1:1, v/v) that were spiked into blank brain tissue homogenate to yield a series of calibration standard samples in the range of 1 ng/mL to 10 µg/mL. All tissue samples were treated identically to the calibration standards. LC‐MS/MS analysis was performed using multiple reaction monitoring for detection of characteristic ions for each drug candidate, additional related analytes, and internal standard. The LLOQ was 1 ng/mL.

#### 2.6.6 Western blot

One third of the mixed‐chopped left hemisphere tissue was weighed and homogenized in a ratio of 1:9 with tissue homogenization buffer (THB; W:V, 1:9). THB homogenization buffer was prepared as follows: 2 mM Tris‐HCl (pH 7.5), 250 mM sucrose, 0.5 mM EDTA, and 0.5 mM egtazic acid with 1x Halt phosphatase/protease inhibitor (Thermo Fisher #78440) and the homogenate was used for western blot (WB). The protein concentration of homogenate was quantified by Pierce Detergent Compatible Bradford Assay Kit (Thermo Fisher #23246) according to the kit instructions. Each sample was mixed with Laemmli Sample Buffer (Bio‐Rad, #1610747) and boiled for 5 minutes at 95°C. Fifteen µg of total protein from each sample was loaded into a 4% to 20% Criterion TGX Precast gel (Bio‐Rad #5671095) or 4% to 20% Mini‐PROTEAN TGX gel (Bio‐Rad # 4568096). Proteins were transferred onto a 0.2 µm nitrocellulose membrane (Bio‐Rad # 1704159), blocked with EveryBlot Blocking Buffer (Bio‐Rad#12010020) for 30 minutes at room temperature, then incubated with the diluted primary antibody solution at 4°C overnight. After overnight incubation, the membranes were washed with TBST (25 mM Tris, pH 7.4, 3.0 mM KCl, 140 mM NaCl, and 0.05% Tween 20) three times for 10 minutes each at room temperature followed by incubation with goat anti‐rabbit IgG StarBright Blue 700 secondary antibody (1:3000; Bio‐Rad #12004161) for 1 hour at room temperature. Protein expression was visualized with ChemiDoc Imaging System (Bio‐Rad). The primary antibodies used for these studies were anti‐PAK1antibody (1:10000; Abcam #ab223849) and anti‐Phospho‐PAK1 (Ser144)/PAK2 (Ser141) antibody (1:1000; Cell Signaling, #2606). Anti‐GAPDH hFAB Rhodamine Antibody (Bio‐Rad 12004167) was used for detecting the internal control protein GAPDH. The OD (optical density) of WB images were quantified by ImageJ software (NIH). Protein expression for each sample was normalized to the GAPDH internal control, and the fold‐change was calculated relative to the average expression levels of age‐ and sex‐matched 5xFAD vehicle‐treated controls for each antibody separately (PAK1, pPAK1); with the ratio of pPAK1/PAK1 quantified as a measure of PAK1 activity.

#### 2.6.7 Enzyme‐linked immunosorbent assay

Plasma and brain Aβ40 and Aβ42, as well as NfL were analyzed by enzyme‐linked immunosorbent assay (ELISA). V‐PLEX Aβ Peptide Panel 1 (4G8) Kit (Meso Scale Diagnostics, LLC #K15199E) was used for Aβ40/Aβ42 detection and S‐PLEX Neurofilament L Kit (Meso Scale Diagnostics, LLC # K151AKGS) was used for NfL analysis, according to the manufacturer's protocols. For brain Aβ40 or Aβ42 analysis, the THB brain homogenates (from WB method as described above) were further fractionated to diethylamine (DEA) soluble and formic acid (FA) soluble extracts in line with the published protocols of Casali and Landreth.^27^ Briefly, THB homogenate was mixed with equal volume of 0.4% DEA solution and centrifuged at 135,000 g for 1 hour at 4°C. The supernatant recovered was the DEA soluble fraction containing the soluble Aβ isoforms. The pellet was subsequently resuspended in FA and centrifuged at 109,000 g for 1 hour at 4°C, resulting in the supernatant containing the insoluble Aβ isoforms. For ELISA, the DEA‐soluble fraction was diluted at 10 times and 40 times and the insoluble fraction was diluted at 40 times and 300 times. The assay plates were read by MESO QuickPlex SQ 120 MM plate reader and analyzed using Discovery Workbench Software (MesoScale Discovery). All samples were run in duplicate and averaged for analysis. Brain concentrations of the soluble and insoluble Aβ were normalized to total protein. All samples from a single plasma collection timepoint were run together on the appropriate MSD plate with the kit provided calibration standards. The calibration standards from each plate and the accuracy and precision between and within runs were evaluated based on the US Food and Drug Administration (FDA)’s M10 Bioanalytical Method Validation guidelines for ligand binding assays.^28^ Calibration standards for which the back‐calculated concentrations exceeded ± 20% (or ± 25% at the upper or lower limits of quantification) of the expected concentration (% recovery) were excluded. Across all plates % recovery ranged from 90% to 120%.

#### 2.6.8 Modified Golgi staining

The right hemisphere was subjected to Golgi staining as described previously^29^ to assess dendrite morphology and dendritic spine density. Briefly, frozen mice brains were rapidly immersed in a modified Golgi–Cox staining solution (developed and provided by Deqiang Jing and Francis Lee, Cornell University) for 9 days at room temperature in the dark. The brains were then incubated in 30% sucrose in dH2O at 4°C for 72 hours, with the solution refreshed after the first 12 hours. Next, 150 µm sections were prepared using a vibratome and mounted onto slides coated with 0.3% gelatin. After a brief drying period, slides were dipped three times in 40% sucrose and air dried for 72 hours in the dark. After dH2O washes, sections were developed using a staining solution, rinsed again, dehydrated through a graded ethanol series, cleared in xylene, and cover slipped with DPX mounting medium.

#### 2.6.9 In vivo dendrite tracing and dendritic spine density quantification

Brain sections stained using the Golgi–Cox method were imaged with a Zeiss brightfield microscope equipped with the Neurolucida software (MBF Biosciences). The Neurolucida software was used to acquire neuron pictures, trace neuronal dendrites, and quantify dendritic spine density. First, the 10x objective was used to identify and focus on brain regions of interest: hippocampus (CA1) or layer V of somatosensory cortex. The selection and identification of these brain regions was done based on the anatomical landmarks in coronal sections according to the Paxinos and Franklin Mouse Brain Atlas (3rd edition). Neurons that were relatively isolated and did not have interference from dendrites and somas of neighboring neurons were selected for performing the dendritic tracing. After a neuron was selected, the 100 × oil immersion objective was used to perform real time dendritic tracing using Neurolucida's live mode. The soma was outlined using the “cell body” option followed by manual tracing of apical and basal dendrites using the respective “dendrite tracing” options. After the tracing was finished, spines were manually marked along the dendrites. A.DAT file was saved for each neuron post completion of dendrite and spine tracing and imported into Neurolucida Explorer, which is used to perform quantitative structural analysis and dendrite length and number of spines data for each neuron is exported. The spine density is calculated by dividing number of spines/dendrite length separately for apical and basal dendrites for each neuron.

### 2.7 Confocal imaging, quantification, and data analysis

The cultured hippocampal neurons were imaged using an oil‐based 63x objective on a Leica Stellaris 5 confocal microscope. The cell nucleus was stained with DAPI and imaged at a wavelength of 405 nm (blue channel), the dendrites stained by MAP2 were imaged at 488 nm wavelength (green channel), and the drebrin‐stained spines were imaged at a wavelength of 561 nm (red channel). Z‐stack images of neurons were acquired at a 1024 × 1024 resolution, a zoom factor of 1.0, and a z‐step size of 0.5 µm. The number of steps for every z‐stack picture of a neuron were determined by the size of the neuron in the z‐plane, to ensure that the entirety of a neuron is captured in the picture. The areas to be imaged were selected systematically, starting from the top‐left region to proceeding to the bottom‐right region of a coverslip. For imaging, preference was given to field of views containing isolated neurons versus clustered neurons.

For quantification of drebrin‐positive spines in vitro, the images were analyzed using Imaris 9.3 software. Each image consisted of one major neuron and the “filaments” option in Imaris was applied to manually trace along the dendrites of every neuron, by placing starting and seed points at the beginning and along the length of the dendrites, respectively. The largest dendrite diameter (i.e., Starting Point) was set at 9.00 µm threshold while the thinnest dendrite diameter (i.e., Seed Point) was set at 1.00 µm threshold. After manually tracing the dendrites, the spines along the dendrites were assessed using the automatic “spine detection” function in Imaris. The thinnest spine diameter was set at 0.80 µm threshold while the maximum spine length was set at 5.00 µm. The spine density (number of spines/dendrite length) was then calculated for each neuron. All image acquisition and quantification were performed in a blind manner. For in vitro quantification of dendrite degeneration, Neurolucida (MBF Biosciences) in the manual mode was used to measure the total dendritic length, numbers of dendrites, and dendritic complexity for each neuron.

### 2.8 Proteomic analyses

A total of 91 brain samples were prepared from mice in this study and analyzed by mass spectrometry proteomics. One third of the mixed‐chopped left hemisphere tissue was homogenized in 8 M urea lysis buffer (8 M urea, 10 mM Tris, 100 mM NaH2PO4, pH 8.5) with HALT protease and phosphatase inhibitor cocktail (Thermo Fisher) and sonicated for three cycles consisting of 5 seconds of active sonication at 30% amplitude, followed by 15 seconds on ice. Samples were then centrifuged for 5 minutes at 15,000 g and the supernatant was transferred to a new tube. Protein concentration was determined by bicinchoninic acid (BCA) assay (Pierce). For protein digestion, 200 µg of each sample was aliquoted and volumes normalized with additional lysis buffer. Samples were reduced with 5 mM DTT at room temperature for 30 minutes, followed by 10 mM iodoacetamide (IAA) alkylation in the dark for another 30 minutes. Lysyl endopeptidase (Wako) at 1:25 (w/w) was added, and digestion allowed to proceed overnight. Samples were then 7‐fold diluted with 50 mM ammonium bicarbonate. Trypsin (Promega) was then added at 1:25 (w/w) and digestion proceeded overnight. The peptide solutions were acidified to a final concentration of 1% (vol/vol) FA and 0.1% (vol/vol) TFA and desalted with a 10 mg HLB column (Oasis). Each HLB column was first rinsed with 1 mL of methanol, washed with 1 mL 50% (vol/vol) acetonitrile (ACN), and equilibrated with 2 × 1 mL 0.1% (vol/vol) TFA. The samples were then loaded onto the column and washed with 2 × 1 mL 0.1% (vol/vol) TFA. Elution was performed with 0.5 mL 50% (vol/vol) ACN. Samples were split into 50 µL and 400 µL aliquots. Residuals from all samples were combined to create a global internal standard pool.

Samples were resuspended in an equal volume of loading buffer (0.1% FA, 0.03% TFA, 1% ACN) and analyzed by LC‐MS/MS. Peptide eluents (1 µg) were separated on a Water's CSH (15 cm × 150 µM internal diameter [ID] with 1.7 µm CSH resin) by a Dionex Ultimate 3000 RSLCnano (Thermo Fisher). Buffer A was water with 0.1% (vol/vol) formic acid, and buffer B was 80% (vol/vol) acetonitrile in water with 0.1% (vol/vol) FA. Elution was performed over a 30 minute gradient. The gradient was from 1% to 99% solvent B. Peptides were monitored on an Orbitrap Fusion Lumos mass spectrometer (Thermo Fisher). Each cycle consisted of one full scan (MS1) performed with an *m*/*z* range of 380 to 985 at a 60,000 resolution at standard settings and 30 data independent scans (DIA) in 1.5 seconds. The higher energy collision‐induced dissociation (HCD) tandem scans were collected at 30% collision energy with an isolation of 10 *m*/*z*, a resolution of 15,000, an AGC setting of 200% normalized AGC target, and a maximum injection time set to 40 ms.

Raw data files were converted to mzML format for downstream processing using ThermoRawFilerParser v.1.4.4.^30^ The data were processed using Data‐Independent Acquisition by Neural Network (DIA‐NN) v.1.9.1.^31^ A spectral library was generated using all canonical mouse proteins from UniProt and SwissProt (accessed September 24, 2024) and contaminants from the Common Repository of Adventitious Proteins (cRAP) database [https://www.thegpm.org/crap/]; these contaminant proteins were excluded from normalization and downstream analysis. DIA‐NN was run using parameters specified as follows: precursor charge was set from 1 to 4 and precursor *m*/*z* was set from 200 to 5000. Enzyme digestion was set to strict trypsin with 1 allowed missed cleavage, peptide length was set from 7 to 50, and peptide *m*/*z* was set from 150 to 3000. The maximum number of variable modifications was set to 3 and methionine oxidation was allowed as a variable modification. Cysteine carbamidomethylation was allowed as a fixed modification. MS1 and MS2 mass accuracy (in ppm) were set using autodetect and the scan window was set using autodetect. The false discovery rate (FDR) threshold was set to 1%. A custom script, taking as input the default DIA‐NN search report file output, was used to generate a sample by gene matrix for use in downstream analysis. Data normalization was performed directly by DIA‐NN; these data are in the PG.MaxLFQ column.

More than 4000 proteins were measured per sample (Figure S6A in supporting information) and most proteins (3223) were detected in all samples (Figure S6B). Most of the intrasample variance was driven by genotype (i.e., 5xFAD vs. WT; 24.3% variance explained) and age cohort (i.e., young vs. aged cohort; 7.3% variance explained; Figure S6C‐D), and the effect of NVS‐PAK1‐1 treatment on proteins specifically altered in 5xFAD mice was assessed (Figure S6E). Differentially abundant proteins were assessed using one‐way analysis of variance (ANOVA) followed by post hoc correction using Tukey honestly significant difference (HSD) as previously described^32^ (Table S3 in supporting information). Gene set enrichment analysis (GSEA) was run with the gseGO function within the clusterProfiler R package([v4.14.6^33^) against the org.Mm.eg.db annotation database (v3.20.0;^34^ Table S4 in supporting information). The logFC‐signed negative log10 adjusted *P* value for each protein was used to rank proteins for GSEA. Overrepresentation analysis (ORA) was run with the gost function from the gprofiler2 R package (v0.2.3^35^). Gene Ontology (GO) terms^36^ that were significantly enriched by either GSEA or ORA were mapped onto the TREAT‐AD biological domains of AD that map existing ontologies onto the AD endophenotypic space (syn25428992.v10^18^).

### 2.9 Statistical analyses

Data were analyzed using GraphPad Prism version 10.0.03. The Shapiro–Wilk and D'Agostino–Pearson tests confirmed that the remaining data followed a normal distribution. For in vitro study, to compare more than two groups, one‐way ANOVA was used for parametric testing when the data were normally distributed, non‐parametric testing with Kruskal–Wallis ANOVA and Dunn post hoc analysis was applied when normality testing did not pass. Sample distributions were compared with the two‐sample Kolmogorov–Smirnov (KS) test. Each figure caption details the number of independent cell culture experiments (*n*), data presentation (mean ± standard error of the mean [SEM]), the statistical tests used, and the significance levels; the following *P* value thresholds were used to determine significance: * *P* < 0.05, ** *P* < 0.01, *** *P* < 0.001, and **** *P* < 0.0001. For in vivo study, data were analyzed a priori within sex, using an unpaired comparisons one‐way ANOVA to evaluate differences in the comparison of three or more groups. Note that during the QC analysis of the data *n* = 4 male mice from the young cohort were excluded from analysis as they were erroneously enrolled into the aged cohort in which treatment was terminated early as described below.

## 3 RESULTS

### 3.1 Small molecule PAK1 inhibitors attenuate oAβ‐ and oTau‐induced spine loss and dendrite degeneration in vitro in a dose‐dependent manner

**FIGURE 1:**
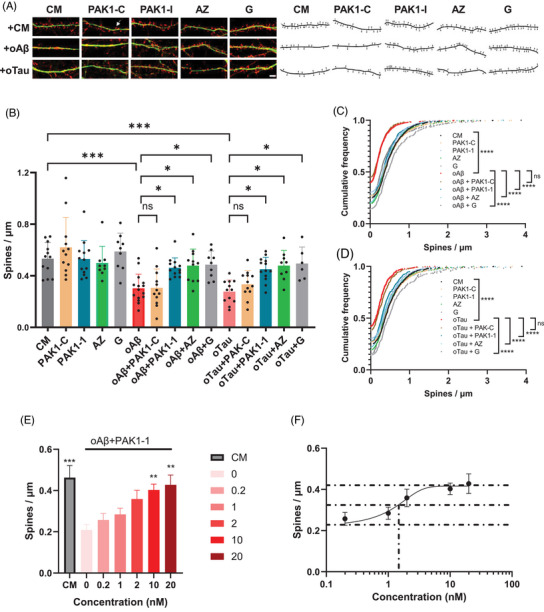
Targeted PAK1 inhibition mitigates oAβ‐ and oTau‐induced synaptic degeneration in vitro, with dose‐responsive protection against oAβ‐mediated spine loss. A–F, 14 DIV mouse hippocampal neurons were treated with either oAβ (1 µM) or oTau (50 nM), with or without PAK1 inhibitors (NVS‐PAK1‐C [PAK1‐C], 10 nM; NVS‐PAK1‐1 [PAK1‐1], 10 nM; AZ13705339 [AZ], 1 nM; G5555 [G], 5 nM), and examined after 24 hours of treatment. The neurons were stained with drebrin (a marker for spines, red) and MAP2 (a marker for dendrites, green), the drebrin‐positive spines were quantified by Imaris in a blinded fashion. All data are presented as mean ± SE. A, Representative confocal images and tracing of drebrin‐positive spines (white arrow indicates a representative spine), scale bar 20 µm. B, Quantitation of number of drebrin‐positive spines per length of a dendrite segment. For oAβ assay, *n*  =  9–15 neurons per condition; for oTau assay, *n* = 6–12 neurons per condition. Kruskal–Wallis with post hoc Dunn multiple comparisons test. C, D, Cumulative frequency, Kolmogorov–Smirnov testing, each group was compared to either oAβ or oTau, no significant difference between Aβ and Aβ+PAK1‐C, oTau, and oTau+PAK1‐C, respectively. E, F, 14 DIV hippocampal neurons were exposed to oAβ and treated with the different concentration of NVS‐PAK1‐1 and examined after 24 hours of treatment. All data are presented as mean ± SE. Dose response graph and curve were show, *n* = 12 neurons per condition. Kruskal–Wallis with post hoc Dunn multiple comparisons test. * *P* < 0.05, ** *P* < 0.01, *** *P* < 0.001, **** *P* < 0.0001. CM, culture medium; DIV, days in vitro; oAβ, oligomeric amyloid beta; oTau, oligomeric tau; PAK1, p21‐activated kinase 1; SE, standard error

Exposure to soluble oAβ or oTau typically results in spine loss and dendrite degeneration in cultured hippocampal neurons at ≥ 14 days in vitro (DIV).^24^, ^37^ In the present study, exposure of 14 DIV hippocampal primary neurons to either oAβ or oTau showed a ≈ 50% decrease (*P* < 0.001) in drebin‐stained spine density (spine counts per µm of dendritic length; Figure 1A,B) as well as significant decreases (*P* < 0.0001) in the cumulative relative frequency of spine density (Figure 1C, D) compared to culture medium (CM). In the presence of PAK1 inhibitors NVS‐PAK1‐1, G5555, and AZ13705339 (referred to as PAK1‐1, G, AZ), both oAβ‐ and oTau‐treated neurons showed significantly higher spine counts (*P* < 0.05) and cumulative relative frequency (*P* < 0.0001) than neurons treated with oligomer species alone. This effect was not reflected in neurons treated with inactive control compound NVS‐PAK1‐C (PAK1‐C).

To further understand the impacts of dosing on spine protection and to establish an effective in vivo regimen for the NVS‐PAK1‐1, we determined its EC_50_ values. The EC_50_, defined as the concentration at which NVS‐PAK1‐1 reduced the degree of oAβ‐ or oTau‐induced loss of spine density by 50%, served as a critical reference for selecting appropriate in vivo doses. We exposed cultured hippocampal neurons to oAβ along with different concentrations of NVS‐PAK1‐1. oAβ significantly (*P* < 0.001) induced spine loss compared to the CM (Figure 1E), confirming earlier findings, and cotreatment with 10 nM and 20 nM NVS‐PAK1‐1 significantly (*P* < 0.01) inhibited spine loss relative to oAβ‐treated neurons. Assessing all concentrations and the resulting dose–response curve (Figure 1F) revealed a clear dose‐dependent spine protection effect and an EC_50_ of ≈ 2 nM, which guided subsequent in vivo studies.

**FIGURE 2:**
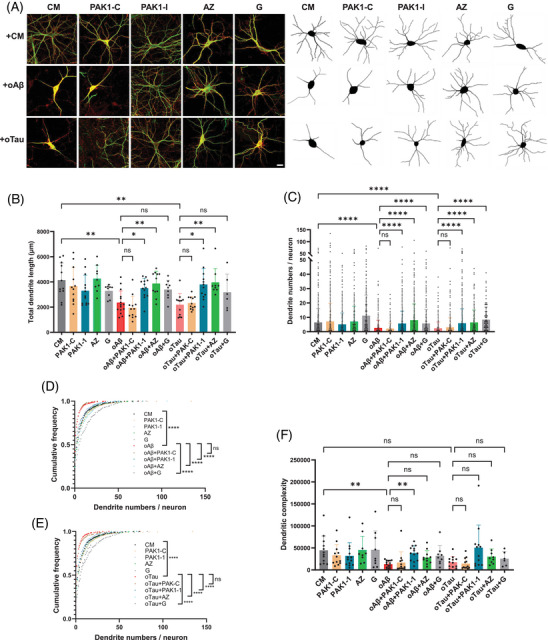
Selective PAK1 inhibitors protect against oAβ‐ or oTau‐induced dendritic degeneration in cultured hippocampal neurons. A–F, 14 DIV hippocampal neurons were treated with either oAβ (1 µM) or oTau (50 nM), with or without PAK1 inhibitors (NVS‐PAK1‐C [PAK1‐C], 10 nM; NVS‐PAK1‐1 [PAK1‐1]), 10 nM; AZ13705339 [AZ], 1 nM; G5555 [G], 5 nM), and examined after 24 hours of treatment. The neurons were stained with drebrin (a marker for spines, red) and MAP2 (a marker for dendrites, green), MAP2‐positive dendrites were traced with Neurolucida under blinded conditions. A, Representative confocal images and tracing of dendrites, scale bar 20 µm. B, Quantitation of total dendrite length per neuron. *n*  =  6–15 neurons per condition, Kruskal–Wallis with post hoc Dunn multiple comparisons test. C, Quantitation of dendrite number per neuron, *n*  =  114–555 dendrites per condition, Kruskal–Wallis with post hoc Dunn multiple comparisons test. D, E, Cumulative frequency for numbers of dendrites, Kolmogorov–Smirnov testing, each group was compared to either oAβ or oTau. F, Quantitation of dendritic complexity. *n*  =  6–15 neurons per condition, Kruskal–Wallis with post hoc Dunn multiple comparisons test. * *P* < 0.05, ** *P* < 0.01, *** *P* < 0.001, **** *P* < 0.0001. CM, culture medium; DIV, days in vitro; oAβ, oligomeric amyloid beta; oTau, oligomeric tau; PAK1, p21‐activated kinase 1

A similar pattern occurred with dendrite degeneration. The total length per neuron of MAP2‐stained dendrites was significantly reduced in neurons exposed to either oAβ or oTau compared to CM (Figure 2A,B, *P* < 0.01). Cotreatment of neurons with NVS‐PAK1‐1 or AZ significantly (*P* < 0.01) preserved dendrite length compared to treatment with either oAβ or oTau alone. Neither G nor PAK1‐C showed a significant dendrite length difference. In terms of dendrite number per neuron (Figure 2C), both oAβ and oTau‐treated neurons had significantly (*P* < 0.0001) fewer dendrites than observed in the CM condition. Cotreatment with NVS‐PAK1‐1, AZ, or G significantly increased dendrite number per neuron (*P* < 0.0001) compared to oAβ or oTau treatment alone. This same pattern was also apparent in cumulative relative frequencies of dendrites per neuron (Figure 2D,E). Last, when examining dendritic complexity (Figure 2F), only oAβ‐treated neurons showed a significant (*P* < 0.01) loss of complexity relative to CM, and only NVS‐PAK1‐1 showed a significant (*P* < 0.01) preservation of complexity relative to oAβ‐treated alone. Taken together, these results demonstrate that NVS‐PAK1‐1 can prevent spine loss and dendrite degeneration in vitro against either toxic oAβ or oTau, and that the protective effect is not present with inactive control compounds. Previous study has found that both G5555 and AZ13705339 inhibit off‐target kinases, and that non‐selective inhibition of PAK1/2 can lead to cardiotoxicity^38^ https://www.optibrium.com/downloads/Resolving_on‐or_off_target_toxicity.pdf). Given NVS‐PAK1‐1 had a spine‐protective effect and is selective for PAK1 inhibition,^22^ it was moved forward as a candidate for in vivo experiments.

### 3.2 The PAK1 allosteric inhibitor NVS‐PAK1‐1 is an orally bioavailable CNS‐penetrant molecule

To enable the translation of these findings from in vitro to in vivo, we conducted an acute PK study in normal CD‐1 mice to evaluate the oral bioavailability and brain exposure of NVS‐PAK1‐1. Initial analysis compared IP and PO for treatment route differences in brain exposure. After 1 hour of pretreatment (10 mg/kg, IP) the concentration of NVS‐PAK1‐1 (mean ± standard deviation, *n* = 3) in brain was 112 ± 14 ng/g while the concentration of NVS‐PAK1‐1 in plasma was 448 ± 65 ng/mL; with a calculated average brain: Plasma ratio = 0.25. No adverse events were observed. PK parameters (Table S5 in supporting information) demonstrate comparative exposure levels in plasma after both IP and oral PO administration. Confirmatory PK assessment of NVS‐PAK1‐1 PO at 1, 10, and 100 mg/kg revealed dose‐ and time‐dependent changes in concentrations of NVS‐PAK1‐1 in plasma and brain (Table 1) estimated by a non‐compartmental model using WinNonlin 8.3. The lowest dose of NVS‐PAK1‐1 evaluated (1 mg/kg, PO) was detected in plasma but was below the limit of quantification (BLOQ < 1 ng/mL) in brain after oral administration (1 mg/kg PO) at 1, 3, and 8 hours post dose (Table S6 in supporting information). Only the highest dose of NVS‐PAK1‐1 evaluated (100 mg/kg, PO) was detectable in the brain at 8 hours.

**TABLE 1:** Plasma and brain PK parameters after PO administration of 1, 10, and 100 mg/kg NVS‐PAK1‐1 (For these studies sampling was conducted at 1, 3, and 8 hours post dose).

		Plasma			Brain	
PK parameters	Unit	1 mg/kg PO	10 mg/kg PO	100 mg/kg PO	10 mg/kg PO	100 mg/kg PO
T_1/2_	h	2.62	1.40	1.50	0.942	1.23
T_max_	h	1.00	1.00	1.00	1.00	1.00
C_max_	ng/mL	8.10	112	2333	33.2	759
AUC_last_	h*ng/mL	27.2	274	4570	57.4	1380
AUC_Inf_	h*ng/mL	31.6	280	4716	67.8	1399
AUC__%Extrap_obs_	%	14.0	2.29	3.08	15.3	1.34
MRT_Inf_obs_	h	3.92	2.10	1.91	1.74	1.56
AUC_last_/D	h*mg/mL	27.2	27.4	45.7	5.74	13.8
AUClast(brain/plasma)	–	–	–	–	0.210	0.302

Abbreviations: NVS‐PAK1‐1, a selective p21‐activated kinase 1 inhibitor; PK, pharmacokinetic; PO, oral administration.

The results established oral bioavailability and brain penetration of NVS‐PAK1‐1, providing critical insights for dose optimization in subsequent studies. In CD‐1 mice, oral dosing achieved systemic and central nervous system exposure exceeding the in vitro EC_50_, section 3.1 above, indicating sufficient concentrations to modulate PAK1 activity in the brain.

### 3.3 The PAK1 allosteric inhibitor NVS‐PAK1‐1 achieves in vivo target engagement in the brain after acute oral administration

**FIGURE 3:**
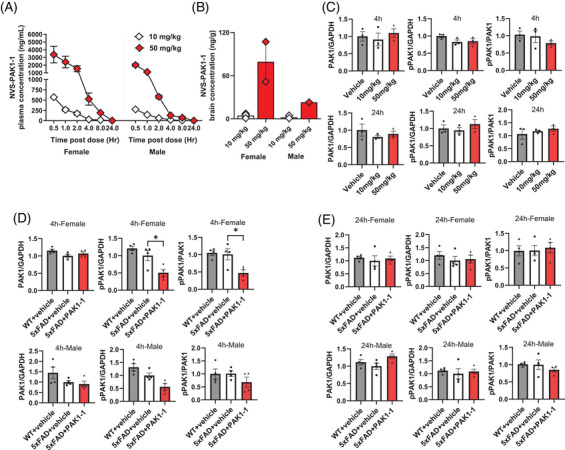
NVS‐PAK1‐1 exhibits brain penetration, bioavailability, and in vivo target engagement in acute oral administration to 5xFAD mice. A–C, A pilot PK study was conducted in female and male 5xFAD mice after oral administration of NVS‐PAK1‐1 at doses of 10 mg/kg or 50 mg/kg. Plasma concentrations were measured at multiple timepoints, while brain concentrations and PAK1 activity were assessed at a single timepoint (4 hours or 24 hours) for each subject. A, Plasma concentration–time profiles of NVS‐PAK1‐1 from serial sampling in male and female 5xFAD mice. Each data point represents plasma concentrations (ng/mL; mean ± SD; *n* = 3–4) of NVS‐PAK1‐1. B, Brain concentration of NVS‐PAK1‐1 in male and female 5xFAD mice at 4 hours post oral administration of 10 or 50 mg/kg of NVS‐PAK1‐1 (ng/g; mean ± SD; *n* = 3). C, Expression levels of PAK1 and pPAK1 in brain tissues were analyzed by western blot after acute oral administration of 10 or 50 mg/kg NVS‐PAK1‐1 (PO). PAK1 activity as measured by the pPAK1/PAK1 ratio was reduced by 25% relative to vehicle‐treated controls at 4 hours post 50 mg/kg NVS‐PAK1‐1 (PO). The reduction in PAK1 activity was absent at 24 hours post dose (mean ± SD; *n* = 3 per dose level). D, Confirmation of PAK1 inhibition by NVS‐PAK1‐1 in aged female and male 5xFAD mice. Acute oral administration of 100 mg/kg NVS‐PAK1‐1 (4 hours post dose, PO) produced reductions in PAK1 activity as measured by the pPAK1/PAK1 ratio relative to vehicle‐treated age‐ and sex‐matched 5xFAD controls (mean ± SD; *n* = 4 per sex per treatment group). The fold changes of PAK1, pPAK1, and pPAK1/PAK1 were compared to sex‐matched 5xFAD vehicle‐treated controls and analyzed by one‐way analysis of variance. The reductions in pPAK1 and pPAK1/PAK1 were greater at 100 mg/kg relative to 50 mg/kg indicative of a dose–response relationship. E, The inhibitory effect of NVS‐PAK1‐1 was absent at 24 hours post dose as measured by expression levels of PAK1, pPAK1, and the pPAK1/PAK1 ratio, and consistent with the lack of detectable levels of NVS‐PAK1‐1 in brain or plasma at 24 hours post administration. * *P* < 0.05, ** *P* < 0.01, *** *P* < 0.001, **** *P* < 0.0001. NVS‐PAK1‐1, a selective p21‐activated kinase 1 inhibitor; PAK1, p21‐activated kinase 1; PK, pharmacokinetic; PO, oral administration; pPAK1, phospho p21‐activated kinase 1; SD, standard deviation

Next, we assessed the PK/PD of PO administered NVS‐PAK1‐1 in 5xFAD mice. Initial pilot PK/PD conducted in aged female and male 5xFAD mice followed NVS‐PAK1‐1 at doses of 10 or 50 mg/kg. Serial plasma concentrations were measured over time, and brain concentrations were assessed at 4 and 24 hours post dose. Dose‐dependent increases in exposure in plasma and brain with appreciable brain concentrations at 4 hours post dose were observed, that were BLOQ at 24 hours post dose (Figure 3A,B). Notably, females exhibited higher brain concentrations of NVS‐PAK1‐1 than males at both 10 and 50 mg/kg doses (Figure 3B). Looking at combined male and female groups, the concentrations of NVS‐PAK1‐1 in the brain at 4 hours post dose corresponded to a reduction of PAK1 activity as supported by a 25 ± 13% reduction in the ratio of pPAK1/PAK1 relative to vehicle‐treated 5xFAD, indicative of target engagement (Figure 3C, top panel). The reduction of PAK1 activity observed at 4 hours post dose was again absent at 24 hours post dose when exposure levels in brain were BLOQ (Figure 3C, bottom panel). Based on the initial pilot PK/PD data and to further explore sex‐specific differences in brain concentration, 100 mg/kg NVS‐PAK1‐1 or vehicle control was administered to sex‐matched cohorts (*n* = 4/per sex/per treatment/per genotype) of female and male, 5xFAD and WT mice with planned timepoints at 4 and 24 hours post‐administration. At 4 hours post dose (Figure 3D), 100 mg/kg NVS‐PAK1‐1 significantly (*P* < 0.05) inhibited PAK1 activity in female 5xFAD mice, resulting in a 54 ± 16% reduction (Figure 3D, top panel) in the ratio of pPAK1/PAK1 relative to vehicle‐treated 5xFAD. In male 5xFAD mice, a non‐significant reduction of 33 ± 24% in the pPAK1/PAK1 was observed (Figure 3D, bottom panel). The effects of NVS‐PAK1‐1 on PAK1 activity observed at 4 hours were absent at 24 hours in both sexes (Figure 3E), aligning with previous PK profiles of NVS‐PAK1‐1. Consistent with known sex differences in pharmacokinetics, females exhibited a greater pharmacodynamic response than males, highlighting potential sex‐based variability in drug efficacy (Figure 3D). Nonetheless, PK/PD identified successful CNS penetration of NVS‐PAK1‐1 in the 5xFAD AD model, and a reduction in brain PAK1 activity, reinforcing potential for further preclinical development.

### 3.4 PD effects of chronic PAK1 inhibition in 5xFAD mice via long‐term PO

Given the successful inhibition of PAK1 activity during acute NVS‐PAK1‐1 administration, we explored the impact of long‐term dosing with the PAK‐1 inhibitor in 5xFAD and WT mice over an extended period. PK/PD analysis indicated a short half‐life of NVS‐PAK1‐1, so evaluating the PD effects of chronic PAK1 inhibition aimed to maximize exposure levels in brain and minimize Cmin:Cmax. Therefore, a dose of 100 mg/kg NVS‐PAK1‐1 (PO) administered twice daily for a 12‐week treatment duration was planned. For these studies, we evaluated treatment effects of chronic PAK1 inhibition on 5xFAD mice, both in aged mice (8–9 months at treatment initiation) during the late stages of amyloid plaque deposition, and in young mice (aged 2–3 months at treatment initiation) during the early stages of amyloid plaque deposition. We evaluated PAK1 activation (pPAK1/PAK1), synaptic integrity (Golgi staining), and AD‐relevant biomarkers to evaluate potential prevention of spine loss in young mice or restoration of synaptic integrity in aged mice with significant amyloid accumulation.

**FIGURE 4:**
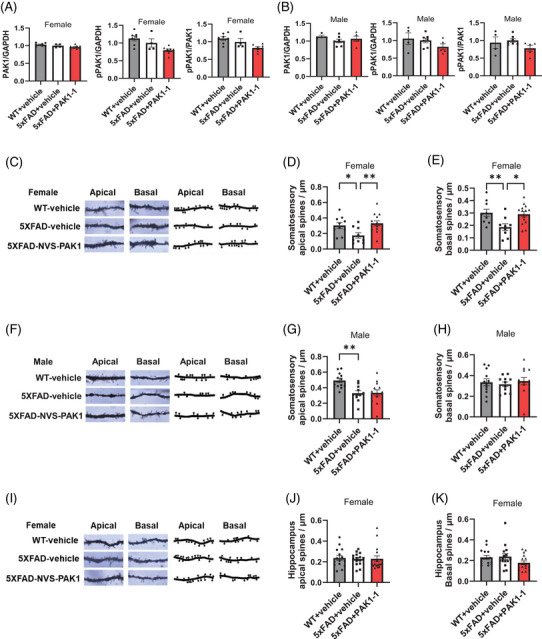
Chronic oral administration of NVS‐PAK1‐1 is associated with reduced spine degeneration in young 5xFAD mice. A–K, 5xFAD female and male mice (2–3 months old) were chronically administered the NVS‐PAK1‐1 via oral administration at a dose of 100 mg/kg, twice daily for 12 weeks. Brains were collected 4 hours after final dose and processed for PAK1 activity assessment and Golgi staining. A–B, PAK1 activity as measured by total pPAK1 expression and by the pPAK1/PAK1 ratio relative to vehicle‐treated age‐ and sex‐matched 5xFAD controls. The fold changes of PAK1, pPAK1, and pPAK1/PAK1 were compared to sex‐matched 5xFAD vehicle treated controls and analyzed by one‐way ANOVA; (A) represents females, and (B) represents males. C–K, Spine density measured using Neurolucida in either the somatosensory cortex or hippocampal CA1 region. C, Representative Golgi staining and tracings of spine numbers per dendrite in the somatosensory cortex of young female mice. Representative Golgi staining images represent a single *z* plane, while Neurolucida tracings were based on multi‐*z* plane visualization. D–E, Quantification of total spine density in the apical and basal dendrites. Statistical analysis was performed using ordinary one‐way ANOVA with Dunn multiple comparisons test, *n* = 9–14 neurons per condition from 5–8 mice. F, Representative Golgi staining and tracings of spine numbers per dendrite in the somatosensory cortex of young male mice. G–H, Quantification of total spine density in the apical and basal dendrites. Statistical analysis was conducted using ordinary one‐way ANOVA with Dunnett multiple comparisons test. *n* = 10–16 neurons per condition from 4–5 mice. I, Representative Golgi staining and tracings of spine numbers per dendrite in the hippocampal CA1 region of young female mice. J–L, Quantification of total spine density in the apical and basal dendrites. Statistical analysis was conducted using Kruskal–Wallis test with Dunn multiple comparisons test. *n* = 15–18 neurons per condition from 5–8 mice. * *P* < 0.05, ** *P* < 0.01, *** *P* < 0.001, **** *P* < 0.0001. ANOVA, analysis of variance; NVS‐PAK1‐1, a selective p21‐activated kinase 1 inhibitor; PAK1, p21‐activated kinase 1; PO, oral administration; pPAK1, phospho p21‐activated kinase 1; WT, wild type

In young 5xFAD mice, chronic treatment with NVS‐PAK1‐1 (100 mg/kg, BID, PO for 12 weeks) trended toward modest reductions of 17 ± 9% and 22 ± 12% in PAK1 activity in female and male mice, respectively, as measured by pPAK1/PAK1 ratio relative to age‐ and sex‐matched vehicle‐treated 5xFAD; collected 4 hours after the final dose (Figure 4A,B). Terminal brain and plasma levels of NVS‐PAK1‐1 after chronic treatment showed a differential NVS‐PAK1‐1 exposure level between sexes, with mean brain concentrations in 5xFAD males of 98.5 ± 34.4 ng/g, which was significantly greater (*P* < 0.01) than in female 5xFAD (44.9 ± 14.1 ng/g). Average plasma concentrations were comparable across sex with the 5xFAD male plasma concentrations of 309.4 ± 104.5 ng/mL not significantly differing from the 5xFAD females (324.4 ± 104.5 ng/mL).

However, in 8‐ to 9‐month‐old 5xFAD mice with significant amyloid deposition, chronic twice daily oral administration of both NVS‐PAK1‐1 and vehicle resulted in significant weight loss (≈ 20%) within the first 31 days of treatment. We also observed an increase in observations of handling‐induced seizures, which was independent of treatment group. Notably, spontaneous seizure activity has previously been reported for 5xFAD mice.^39^ These events were not related to the drug treatment as they occurred at a similar prevalence across both vehicle‐ and drug‐treated groups, likely a stress‐related effect of twice daily restraint and oral gavage in aged 5xFAD mice. Out of an abundance of caution, the planned 12‐week treatment for the aged cohort was terminated early at 31 days post‐treatment. Chronic 31‐day treatment with the NVS‐PAK1‐1 had no effect on PAK1 activity in either aged female (Figure S7 in supporting information, top panel) or aged male (Figure S7, bottom panel) 5xFAD mice. Brain NVS‐PAK1‐1 concentrations in aged male and female 5xFAD mice were 49.1 ± 17.6 and 58.3 ± 26.1 ng/g, respectively, while plasma NVS‐PAK1‐1 concentrations were 139.7 ± 27.9 and 354.2 ± 137.8 ng/mL.

### 3.5 Chronic treatment with NVS‐PAK1‐1 mitigates somatosensory spine loss in 6‐month‐old female 5xFAD mice

Because dysregulated PAK1 activity in AD is associated with synaptic malfunction and dendritic spine loss,^15^ we determined whether the inhibition of PAK1 activity observed in our study mitigates spine loss induced by Aβ deposition in 5xFAD mice. Previous studies reported that in 5xFAD female mice at 6 months, spine density is reduced in the basal but not the apical layer of the somatosensory cortex.^40^, ^41^ Our Golgi staining and quantitative analysis of somatosensory cortical dendritic spines demonstrated a significant (*P* < 0.05; *P* < 0.01) reduction in apical and basal spine density in vehicle‐treated 5xFAD female mice compared to WT controls. Chronic treatment with NVS‐PAK1‐1 (100 mg/kg, BID, PO for 12 weeks), from 3 to 6 months of age, prevented the loss of both apical (*P* < 0.01; Figure 4C,D) and basal (*P* < 0.05; Figure 4C,E) dendritic spine density in females. This prevention of spine loss correlates with the inhibition of PAK1 by the NVS‐PAK1‐1 in female mice (Figure 4A), suggesting that PAK1 inhibition counteracts synaptic degeneration associated with aberrant PAK1 activation. In young male mice, apical spine density was also significantly (*P* < 0.01) decreased in 5xFAD vehicle‐treated mice compared to WT controls (Figure 4F,G), with basal spine density demonstrating no significant difference (Figure 4F,H). In both apical and basal spine density, chronic oral administration of NVS‐PAK1‐1 showed no significant difference, despite the observed trend of reduced of PAK1 activation in male NVS‐PAK1‐1 treated mice (Figure 4B), suggesting a sex‐specific efficacy.

Previous studies examining hippocampal CA1 spine loss in 5xFAD mice at 6 months have reported conflicting findings, with one study reporting a significantly reduced spine density in the 5xFAD mice compared to WT control^42^ and another reporting no significant difference.^41^ Our analysis revealed no significant difference in the spine density of CA1 pyramidal neurons between 5xFAD and WT female mice (Figure 4I‐K), consistent with the findings of Crowe et al.^41^ The lack of detectable differences in spine density between 5xFAD and WT mice at 6 months of age made it unfeasible to determine whether the PAK1 inhibitor NVS‐PAK1‐1 could prevent spine loss in CA1 pyramidal neurons (Figure 4I‐K). Taken together, these results show promising prevention of synaptic loss in a particularly vulnerable region of the brain in 5xFAD female mice.

### 3.6 NVS‐PAK1‐1 affects proteome response to 5xFAD mice

**FIGURE 5:**
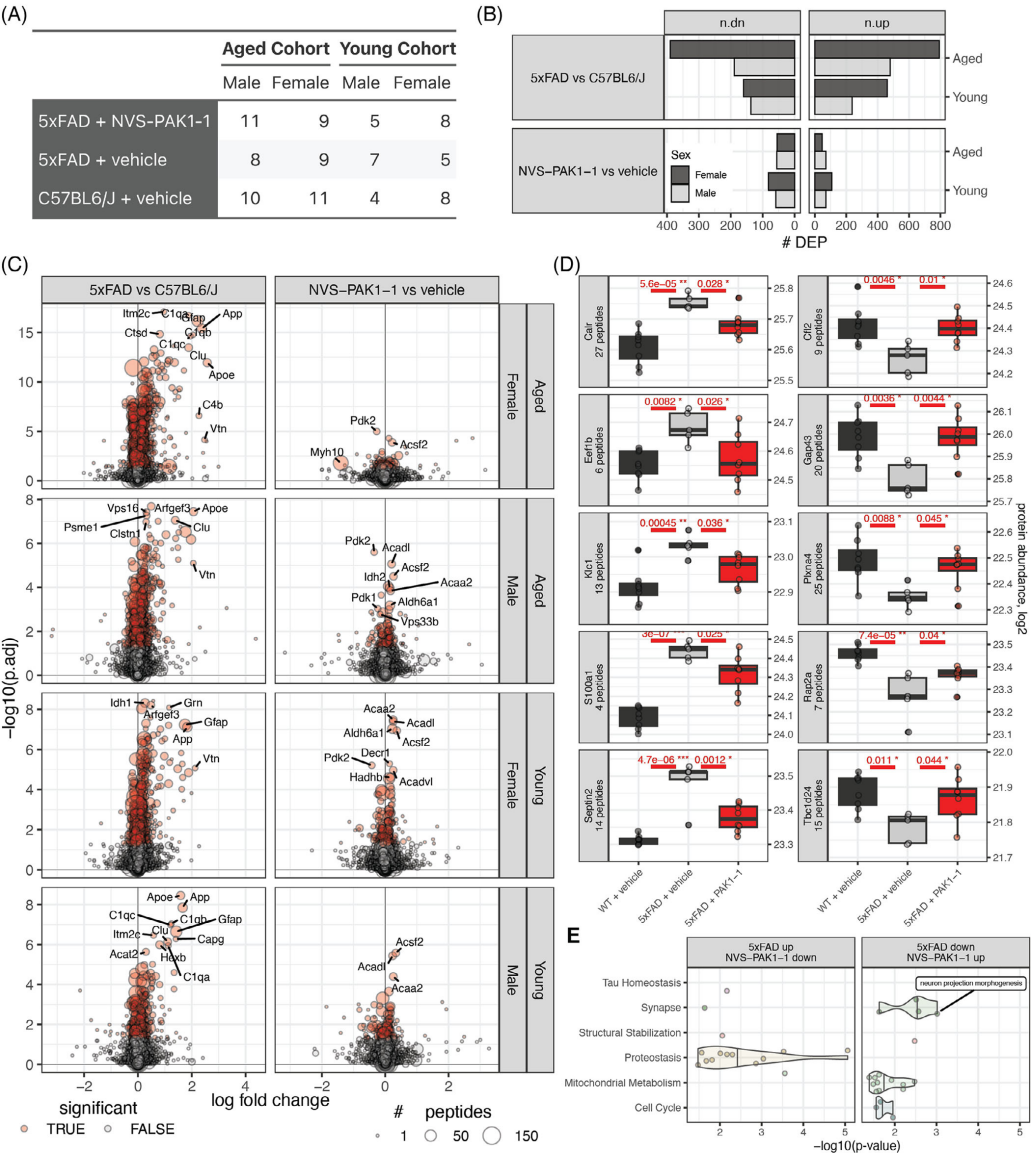
Chronic oral administration of NVS‐PAK1‐1 in 5xFAD mice shows differential expression of proteins, partially reversing young female 5xFAD spine‐related pathology. A–E, Treatment with twice daily oral administration of NVS‐PAK1‐1 (100 mg/kg or vehicle control, BID, PO) was initiated in young (2–3 months old) or aged (9‐month‐old) female and male 5xFAD mice and compared to vehicle‐treated C57BL6/J (WT) littermate controls and 5xFAD vehicle controls. Terminal brain and tissue samples were collected at 4 hours post dose on the final day of treatment which was at 6 months of age (young) or 10 months of age (aged; 30‐day treatment). A, Age and sex demographics of the cohort. B, Total numbers of significantly differentially expressed (*P*adj < 0.05) proteins in each group using analysis of variance followed by post hoc correction using Tukey honestly significant difference. C, Volcano plots of differentially expressed proteins by log fold change in each group. Significant proteins are colored red, and circle size is scaled to number of peptides measured. D, Differential expression of selected proteins in the young cohort female mice that are significantly upregulated in 5xFAD versus WT and downregulated in NVS‐PAK1‐1 treated 5xFAD versus vehicle treated 5xFAD (left) and those that are significantly downregulated in 5xFAD versus WT and upregulated in NVS‐PAK1‐1–treated 5xFAD versus vehicle‐treated 5xFAD (right). E, GO term over‐representation results grouped into TREAT‐AD biological domains for the same young female mice as in (D). Individual circles represent significant (*P*adj < 0.05) GO terms, and violins represent distributions of terms within each biological domain. BID, twice daily; GO, Gene Ontology; NVS‐PAK1‐1, a selective p21‐activated kinase 1 inhibitor; PO, oral administration; TREAT‐AD, Target Enablement to Accelerate Therapy Development for AD; WT, wild type

To better understand the effects of PAK1 inhibition on the brains of 5xFAD mice, we surveyed the proteomic abundance changes to chronic NVS‐PAK1‐1 treatment cohorts as described above. In total we measured the proteomes from 91 animals over two age cohorts, and three arms: 5xFAD treated with NVS‐PAK1‐1, 5xFAD treated with vehicle, and vehicle‐treated WT littermate controls treated with vehicle (Figure 5A). Differential expression analysis focused on the contrasts between vehicle‐treated 5xFAD and WT, as well as between NVS‐PAK1‐1‐ and vehicle‐treated 5xFAD mice, within each age and sex cohort.

For the comparison between vehicle‐treated 5xFAD and WT mice (5xFAD vs. WT), more proteins were found to be significantly differentially expressed in the aged cohort (1183 in females and 694 in males) than in the young cohort (621 in females and 374 in males), and more in the females than the males (Figure 5B, Table S3). This is consistent with the progressive accumulation of neuropathology in aging 5xFAD model animals.^43^, ^44^ Among differentially expressed proteins, there was strong upregulation of proteins commonly associated with Aβ plaques and neuroinflammation (e.g., App, apolipoprotein E [apoE], Gfap, C1qa, Ctsd, etc.; Figure 5C, left panels). Comparing vehicle‐treated versus NVS‐PAK1‐1‐treated 5xFAD mice, relatively few proteins were significantly differentially expressed in response to NVS‐PAK1‐1 treatment; interestingly, there were more proteins significantly differentially expressed in the young cohort (190 in females and 129 in males) than in the aged cohort (101 in females and 134 in males), particularly in young females (Figure 5B, Table S3). This could be due to either a longer period of NVS‐PAK1‐1 treatment (3 months in the young cohort vs. 1 month for the aged) or to the aged cohort being more recalcitrant to the drug effect, but was consistent with a lack of PAK1 inhibition (Figure S7). Among the NVS‐PAK1‐1 effect differentially expressed proteins we noted the upregulation of many enzymes acting on acyl‐CoA (e.g., Acsf2, Acadl, Acaa2, Acadm, Acadvl, etc.) as well as the downregulation of several pyruvate dehydrogenase kinases (e.g., Pdk1, Pdk2, and Pdk3; Figure 5C, right panels), which are notable interactors with group I PAKs, including via the PDK1‐Akt pathway regulating actin reorganization.^45^, ^46^

For a more comprehensive understanding of the functional implications of differential protein expression, we conducted GSEA of GO terms and subsequent stratification into TREAT‐AD AD‐relevant biological domains.^18^ For 5xFAD versus WT, a positive normalized enrichment score (NES) indicated enrichment of upregulated proteins in GO terms within the immune response, proteostasis, apoptosis, and amyloid precursor protein (APP) metabolism domains in all cohorts while a negative NES indicated enrichment of downregulated proteins in GO terms within the mitochondrial metabolism domain (Figure S8A, Table S4 in supporting information). GO terms from the synapse, endolysosome, lipid metabolism, and structural stabilization domains are split: some terms within each domain have a positive NES and others have a negative NES. For the synapse domain, many more GO terms have negative NES values indicating broad reductions in synaptic function across both age cohorts and sexes. Those with a positive NES value involve synaptic turnover (e.g., “synapse pruning”) and regenerative processes (e.g., “positive regulation of axon regeneration”). These results are consistent with previous descriptions of the 5xFAD model indicating increased neuroinflammatory response and synaptic dysfunction.^47^, ^48^ GSEA identified fewer significantly enriched GO terms for NVS‐PAK1‐1 treatment effect (NVS‐PAK1‐1 treated 5xFAD vs. vehicle‐treated 5xFAD) in both age cohorts (Figure S8A‐B, Table S4). The most consistent enrichments across cohorts include positive NES for terms, indicating enrichment of upregulated proteins, within the lipid metabolism domain, including terms related to “lipid catabolic process” and “fatty acid metabolic process,” as well as mitochondrial metabolism in both young males and young females, including “mitochondrial membrane organization.” For the younger cohort females in particular, there was a negative NES for terms within the structural stabilization and proteostasis domains reflecting downregulated proteins, including the “protein maturation” and “actin filament organization” terms, the latter of which is consistent with the function of PAK1 in regulating actin dynamics.^9^, ^46^ There was also a negative NES for terms within the synapse domain, including terms such as “dendrite” and “postsynapse organization,” particularly in aged males and to a lesser extent in females of both ages. Because most of the observed PAK1 inhibition was in the young cohorts, the broad domain proteomic responses to NVS‐PAK1‐1 in vivo are an increase in the expression of proteins involved in lipid and mitochondrial metabolism, and decreased expression of proteins involved in structural stabilization and proteostasis.

Finally, we sought to understand the effect of NVS‐PAK1‐1 treatment on proteins that are specifically affected by the 5xFAD model (Figure S6E). For this comparison we considered any protein that is significantly differentially expressed at an adjusted *P* value ≤ 0.05 in either cohort. We found that of the proteins differentially expressed in both 5xFAD and NVS‐PAK1‐1 treatment contrasts (*n* = 233), there was a significant overlap of proteins that were both up in 5xFAD females and down in NVS‐PAK1‐1 females (*n* = 63, Fisher *P* value = 1.7 × 10^−12^, odds ratio [OR] 3.66), and proteins that were down in 5xFAD females and up in NVS‐PAK1‐1 females (*n* = 22, Fisher *P* value = 0.026, OR 1.67). For the male mice, there was a significant overlap of proteins that are both down in 5xFAD males and up in NVS‐PAK1‐1 males (*n* = 24, Fisher *P* value = 2.34 × 10^−6^, OR 3.43), but the overlap of proteins that were both up in 5xFAD males and down in NVS‐PAK1‐1 males was not significant (*n* = 20, Fisher *P* value = 0.055, OR 1.57). The 63 proteins that were significantly up in 5xFAD versus WT females and were also significantly down in NVS‐PAK1‐1 versus vehicle females included Calr, Eef1b, Klc1, S100a1, and Septin2 (Figure 5D, left column; Table S7 in supporting information). These proteins were significantly over‐represented among GO terms in the proteostasis domain, including the “protein folding” term (*P* value = 8.7 × 10^−6^; Figure 5E, left). Several of these proteins have been implicated in functions related to dendritic spine biology including Klc1, which is involved in the trafficking of proteins to dendrites,^49^ and Eef1b, which controls actin dynamics through regulation of the local translation in dendritic compartments.^50^ There were 22 proteins with significantly lower abundance in 5xFAD and increased abundance in NVS‐PAK1‐1 and they were significantly over‐represented among GO terms in the synapse domain, including the “neuron projection morphogenesis” term (*P* value = 0.00096; Figure 5E, right). Several of these proteins, including Cfl2, Gap43, Plxna4, Hprt1, and Tbc1d24, among others (Figure 5D, right column; Table S7), are functionally linked to the genesis, maintenance, and functioning of dendritic spines. Cfl2, or cofilin 2, is involved in the regulation of actin dynamics that influence spine morphology.^51^, ^52^ Gap43, or growth‐associated protein‐43, is a major component of growth cones and is also involved in regulation of actin cytoskeletal dynamics.^53^ Plxna4, or Plexin A4, is a semaphorin coreceptor and signaling these proteins regulate intracellular actin cytoskeletal dynamics involved in guidance and morphogenesis.^54^, ^55^ Tbc1d24 is a protein that interacts with Arf6 and has been shown to regulate neurite morphology and dendritic arborization.^56^ Hprt1, or hypoxanthine phosphoribosyltransferase 1, is an enzyme involved in the generation of purine nucleotides through salvage, and mutations in the human ortholog of the *Hprt1* gene are causal for Lesch–Nyhan syndrome, which presents with an array of neurological and cognitive phenotypes.^57^ Knockdown of *Hprt1* in a human NT2 cell line has been associated with decreased neurite outgrowth.^58^ Thus, there is a shared response among proteins that are specifically affected by the 5xFAD model and NVS‐PAK1‐1 treatment, and several of the proteins that are differentially expressed in opposing directions indicate putative mechanistic links to explain the observed preservation of spine density in 5xFAD females after NVS‐PAK1‐1 treatment.

### 3.7 NVS‐PAK1‐1 does not affect plasma NfL or Aβ levels in 5xFAD mice

To inform the translatability of these findings to future studies, we also profiled plasma NfL and Aβ levels in the chronically treated young and old cohorts from above. NfL is a structural protein released into the bloodstream when neurons are damaged or degenerated.^59^ Elevated plasma NfL levels have been associated with neurodegeneration and cognitive decline in AD. Research indicates that plasma NfL concentrations increase in AD, even in its prodromal stages, and correlate with disease severity.^60^ Previously, Andersson et al.^61^ found that plasma NfL levels increased with age in 5xFAD mice. Similarly, abnormal plasma Aβ levels and plasma Aβ42/Aβ40 ratio are closely associated with AD pathology. Alteration of plasma Aβ42/Aβ40 ratio may reflect changes in amyloid processing and deposition.^62^ Several studies have reported that plasma Aβ40 and Aβ42 levels as well as Aβ42/Aβ40 ratio are elevated in early‐onset familial AD^63^ and 5xFAD mice.^44^

**FIGURE 6:**
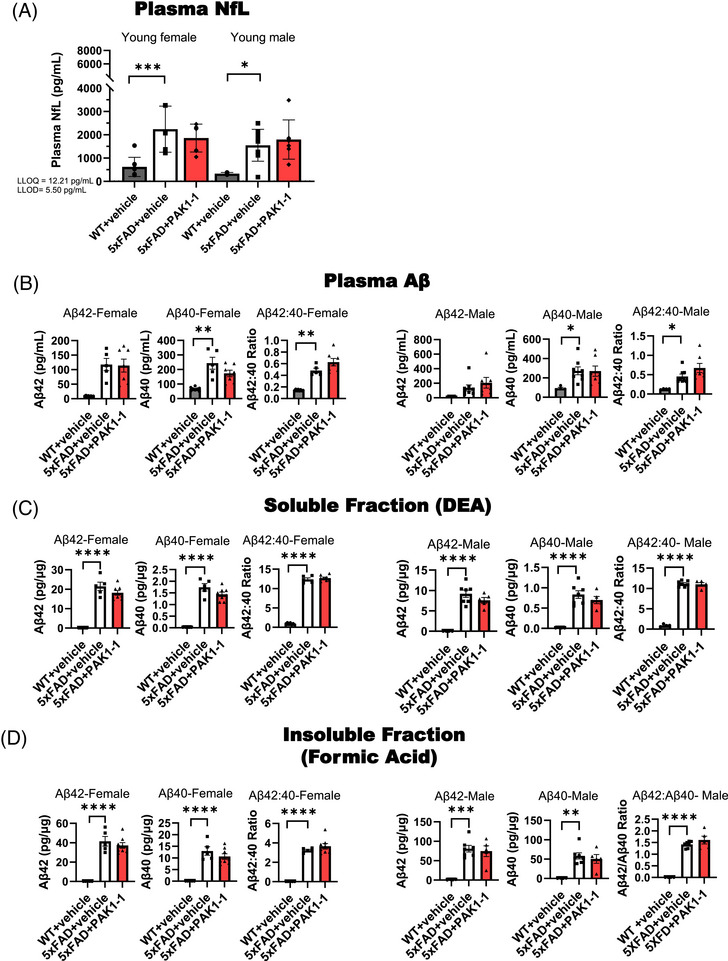
Chronic oral administration of NVS‐PAK1‐1 does not alter plasma concentrations of Aβ40, Aβ42, NfL, or soluble or insoluble brain Aβ40 or Aβ42 in young 5xFAD mice. A–D, Treatment with twice daily oral administration of NVS‐PAK1‐1 (100 mg/kg or vehicle control, BID, PO) was initiated in young (2–3 months old) female and male 5xFAD mice and compared to vehicle‐treated WT littermate controls and 5xFAD vehicle controls. Terminal brain and tissue samples were collected at 4 hours post dose on the final day of treatment which was at 6 months of age. A, As expected, there was an increase of plasma NfL in 5xFAD mice relative to age‐ and sex‐matched vehicle‐treated WT littermate controls. There was no significant difference in NVS‐PAK1‐1–treated 5xFAD mice relative to vehicle‐treated 5xFAD. B–D, Plasma Aβ40 and Aβ42 (B), soluble fraction (C), and insoluble fraction of brain (D). Aβ40 and Aβ42 were quantified by Meso Scale Discovery enzyme‐linked immunosorbent assay are illustrated for young females (left) and young males (right). Vehicle‐treated 5xFAD mice demonstrated the expected increases in Aβ40, Aβ42, and Aβ42/40 relative to vehicle‐treated age‐ and sex‐matched WT littermate controls. There was no significant difference in NVS‐PAK‐1 treatment in 5xFAD mice relative to age‐ and sex‐matched 5xFAD vehicle‐treated controls. Data are presented as mean ± standard deviation (*n* = 4–8 per sex per age per treatment). * *P* < 0.05, ** *P* < 0.01, *** *P* < 0.001 by one‐way analysis of variance with Šídák multiple comparisons test. Aβ, amyloid beta; BID, twice daily; DEA, diethylamine; NfL, neurofilament light chain; NVS‐PAK1‐1, a selective p21‐activated kinase 1 inhibitor; PO, oral administration; WT, wild type

Consistent with previous reports, plasma NfL (Figure 6A, Figure S9A in supporting information), Aβ42, Aβ40, and Aβ42/Aβ40 ratio (Figure 6B–D, Figure S9B‐D), were all significantly elevated in 5xFAD mice compared to age‐ and sex‐matched vehicle‐treated WT littermate controls in all cohorts, with an age‐related increase. Chronic treatment with NVS‐PAK1‐1 did not significantly lower plasma levels for any marker in young, aged, male, or female cohorts of 5xFAD mice. Amyloid accumulation in the brain, both soluble (DEA) and insoluble (FA) fractions were also measured, and results mirrored Aβ plasma values. These findings indicate that neither NfL nor Aβ plasma biomarkers are likely to detect any NVS‐PAK1‐1 treatment‐related changes, at least in 5xFAD mice. Interestingly, because NVS‐PAK1‐1 appears to act independently of Aβ accumulation, PAK1 inhibition may be a promising strategy for mitigating synaptic deficits in AD without directly altering amyloid deposition.

## 4 DISCUSSION

Our findings support the hypothesis that PAK1 inhibition preserves dendritic spine integrity in AD‐relevant models and provides neuroprotective benefits by stabilizing synaptic architecture. Given that synapse loss is one of the strongest correlates with cognitive decline in AD, interventions that protect dendritic spines could have significant implications for modifying disease progression. In this context, the selective allosteric PAK1 inhibitor NVS‐PAK1‐1 offers promising therapeutic potential. In vitro experiments demonstrated that NVS‐PAK1‐1 conferred resilience to dendritic spines against the toxicity of oAβ and oTau, with an EC_50_ of 2 nM. These results suggest that PAK1 inhibition mitigates synaptic vulnerability to toxic oligomeric species, highlighting its potential as a strategy to preserve synaptic function in early‐stage AD.

In line with the benefits of NVS‐PAK1‐1 on oAβ, the 5xFAD mouse model was specifically selected for these studies. Although 5xFAD mice have several limitations, they develop robust amyloid pathology by 6 months of age, have been well characterized by our laboratories, and were appropriate to expand on the in vitro studies that investigated PAK1 effects on oAβ with a focus on target engagement.

PK assessments confirmed that NVS‐PAK1‐1 is orally bioavailable and achieves brain levels above its in vitro EC_50_. In 5xFAD mice, the compound displayed dose‐dependent brain penetration and PAK1 inhibition, as evidenced by reduced PAK1 autophosphorylation (pPAK1 levels). Importantly, in vivo studies demonstrated that oral administration of NVS‐PAK1‐1 effectively inhibited PAK1 activity and prevented spine density loss in 5xFAD female mice without altering amyloid pathology. These findings indicate that PAK1 inhibition acts independently of amyloid clearance, potentially complementing anti‐amyloid AD therapies. This distinction is critical, as current amyloid‐targeting therapies have yielded limited clinical benefit, underscoring the need for alternative strategies that address synaptic resilience.

To elucidate the molecular mechanisms underlying the effects of PAK1 inhibition, we performed brain proteomic profiling of NVS‐PAK1‐1 treated 5xFAD mice across two age cohorts. As expected, vehicle‐treated 5xFAD mice displayed proteomic signatures consistent with AD progression, including upregulation of App, apoE, Gfap, and C1qa, along with enrichment of immune response, proteostasis, lipid metabolism, apoptosis, and APP metabolism pathways. These alterations reflect hallmark features of 5xFAD pathology.^47^, ^48^ Looking at NVS‐PAK1‐1 treatment versus vehicle, the proteomes from females in the younger cohort showed the largest number of changes, likely due to full‐term drug administration and higher target engagement: successful PAK1 inhibition and dendritic spine preservation. Of the strongest changes after NVS‐PAK1‐1 treatment, downregulation of pyruvate dehydrogenase kinases (Pdk1‐3), which are known group I PAKs interactors involved in regulation of actin dynamics and spine formation via the PDK1‐Akt pathway,^45^, ^46^, ^64^ suggested potential mechanistic effects. However, focusing on differentially expressed proteins in the young female cohort, proteins with opposing directions of effect for 5xFAD and NVS‐PAK1‐1 showed a remarkably consistent enrichment of synapse, proteostasis, and mitochondrial metabolism–related changes. Twenty‐two proteins—including Cfl2, Gap43, Plxna4, Hprt1, and Tbc1d24 among others—were significantly down in 5xFAD and up with NVS‐PAK1‐1 treatment, significantly over‐represented in synapse domain terms like “neuron projection morphogenesis” (*P* value = 0.00129), and are functionally linked to the genesis, maintenance, and functioning of dendritic spines.^53^, ^54^, ^55^, ^56^ Cofilin 2 (Cfl2) is directly downstream of PAK1 in the PAK1 → GTPase → LIMK → cofilin signaling cascade^9^ and contributes to actin dynamics and spine protection due to altered PAK1 activity.^51^, ^52^ A likely critical and relevant parallel pathway consists of the RHOA–ROCK2–LIMK1–cofilin signaling cascade in which elevated ROCK2 activity promotes actin disruption and synaptic loss.^65^ Crosstalk between the PAK1 and ROCK2 pathways may further exacerbate cytoskeletal instability and spine degeneration in AD. The 63 proteins that were reversed—up in 5xFAD and down with NVS‐PAK1‐1 treatment—included Calr, Eef1b, Klc1, S100a1, and Septin2. These proteins were significantly over‐represented in the proteostasis domain including terms like “protein folding” (*P* value = 8.3 × 10^−6^) yet several of these proteins are also implicated in functions related to dendritic spine biology including Klc1^49^ and Eef1b.^50^ These findings support the mechanism of PAK1 inhibition‐based spine preservation and were mostly not significant in the mouse cohorts showing little or no PAK1 inactivation. Further findings included broader upregulation of lipid catabolism and mitochondrial metabolism, including mitochondrial enzymes (e.g., Acsf2, Acadl, and Acaa2) with NVS‐PAK1‐1 treatment across all cohorts suggesting a metabolic rebalancing toward fatty acid oxidation and mitochondrial efficiency may help mitigate metabolic dysfunction common in AD, though the direct connection to PAK1 inhibition is uncertain. Together, these proteomic results support a model in which PAK1 inhibition promotes spine resilience through coordinated effects on synaptic structure, cytoskeletal remodeling, and potentially metabolic support.

One of the largest limitations of this study was the unanticipated halt to chronic dosing in the aged cohort, limiting our ability to assess spine‐protective effects in an advanced stage of disease progression. As noted, this was also experienced by the 5xFAD vehicle group and is more likely due to the stress of the oral‐gavage twice‐daily dosing on the mice, which may not generalize to other mouse models or translate to clinical oral administration. Still, some of the proteomic trends we saw, such as lipid metabolism, showed consistent effects even in the older cohorts, suggesting that despite the curtailed 31‐day treatment and adverse impact, NVS‐PAK1‐1 was conferring some effect. Further study would be needed in a different model to determine any beneficence. Additionally, we noted an apparent sex‐specific efficacy effect, with males showing a non‐significant trend in PAK1 activity inhibition and no significant spine rescue. Previous studies have also noted that 5xFAD pathology itself is sex specific, with females exhibiting more aggressive disease progression than males, which may be related at least in part by the Thy‐1 promoter, which has an estrogen response element.^43^, ^44^ In our experiments, young males showed less consistent 5xFAD‐associated spine loss, with significant reductions only in apical dendrites. Although plasma and brain concentrations of NVS‐PAK1‐1 in male cohorts reached comparable levels as those in females, we were unable to explore whether a higher dose, longer dose duration, or slightly older males would result in a significant inhibition of male PAK1 activity, and a comparable spine‐protective effect.

Despite this, our findings are also consistent with prior studies showing that PAK1 inhibition benefits neuronal structure and function across a spectrum of neurodevelopmental and neurodegenerative conditions. In FXS models, PAK1 inhibitors restored cofilin signaling, balanced NMDA/AMPA receptor expression, and improved sensory processing.^66^ Morphometric analyses revealed that inhibiting PAK1 activity with FRAX486 (a non‐selective PAK1 inhibitor) enhanced neurite outgrowth, increased synaptic complexity and the number of intersections per Sholl ring, promoted the formation of new processes, and stimulated the loss of processes in a cellular model of Down syndrome.^67^ In the hippocampus of Cdkl5‐Het mice, treatment with FRAX486, ameliorated spine maturation deficits, enhanced PSD‐95 expression, and normalized aberrant PAK phosphorylation at sites essential for cytoskeletal remodeling.^68^ Beyond neurodevelopmental disorders, PAK1 inhibition has demonstrated neuroprotective effects in injury models. Romidepsin, a dual inhibitor of PAK1 and HDAC approved by the FDA in 2009 for the treatment of cutaneous T‐cell lymphoma, has been shown to mitigate spinal cord injury (SCI)‐induced dendritic spine dysgenesis and alleviate hyperreflexia.^69^ Additionally, romidepsin treatment prevented injury‐induced spine loss and restored mature spine morphology in burn injury models.^70^, ^71^ Similarly, inhibition of PAK1 using IPA‐3 (a PAK1 inhibitor) reduced spinal cord edema, enhanced neuronal survival, and decreased apoptosis after SCI.^72^

The translational potential of PAK1 inhibitors is further supported by oncology clinical trials evaluating their safety and pharmacokinetics.^23^ Notably, PAK1 inhibition via NVS‐PAK1‐1 demonstrates broad therapeutic promise, not only by attenuating aberrant signaling in cancer and neurodegeneration but also by enhancing immune responses, promoting epithelial differentiation in squamous cell carcinoma, and synergizing with chemotherapy to suppress tumor growth and metastasis in triple‐negative breast cancer.^73^, ^74^ Given the role of synaptic dysfunction in AD progression, future studies should investigate the impact of PAK1 inhibition on cognitive outcomes, as well as potential interactions with other AD‐related pathways and other neurodegenerative disease models. 5xFAD are not ideal for studying cognitive function as their hyperactivity is a confound for proper interpretation of mouse behavioral measures, and their rapid and progressive amyloid deposition well before an analogous human age of onset of AD limits their translational relevance; thus, our present studies focused on target engagement and translationally relevant proteomic analyses.^47^, ^75^ Additionally, longer term studies will be necessary to assess whether prolonged PAK1 inhibition can sustain synaptic integrity and provide lasting functional benefits. Understanding the broader implications of PAK1 modulation on neuronal networks, including potential compensatory mechanisms, will be essential for the development of clinically viable therapies.

In conclusion, our data demonstrate that PAK1 inhibition via NVS‐PAK1‐1 protects dendritic spine integrity in AD‐relevant models and modulated multifaceted proteomic changes that counteract disease‐associated synaptic and metabolic dysfunction. By preventing spine loss, supporting synaptic proteins, and enhancing mitochondrial and lipid metabolism, NVS‐PAK1‐1 stabilizes synaptic architecture, a critical determinant of cognitive resilience. These findings position PAK1 as a promising amyloid‐independent therapeutic target for early intervention in AD, with the potential to preserve cognitive function through the maintenance of synaptic integrity.

## AUTHOR CONTRIBUTIONS

Study design, experimental procedures and experiment execution, omics and other data analysis, manuscript preparation: Tao Yang, Hasi Huhe, Alison D. Axtman, Gregory A. Cary, Stacey J. Sukoff Rizzo, and Frank M. Longo. Performance of experiments: Tao Yang, Hasi Huhe, Sean‐Paul Williams, Yeonglong Albert Ay, Sukhneet Kaur, Zachary W. Davis‐Gilbert, Gregory A. Cary, Carolyn Paisie, Jacob L. Capener, David H. Drewry, Mohammad A. Hossain, Hans J. Oh. Additional data analysis and study contributions: Jesse Wiley, Ranjita Betarbet, Haian Fu, Duc Duong, Nicholas T. Seyfried, Karina Leal, Robert R. Butler III, Gregory W. Carter, Aled Edwards, and Allan I. Levey. All authors edited and approved the final version of the manuscript.

## CONFLICT OF INTEREST STATEMENT

No conflicts are declared. Author disclosures are available in the Supporting Information.

## CONSENT STATEMENT

As no human subjects were involved, consent was not required.

## ETHICS STATEMENT

Prior to study initiation, all in vivo experiments were approved by the University of Pittsburgh and Stanford University Institutional Animal Care and Use Committees (IACUC) with strict adherence to the Guide for the Care and Use of Laboratory Animals (NIH Publication No. 85‐23, revised 2011), and in line with ARRIVE guidelines.^26^

## DATA AVAILABILITY/CODE AVAILABILITY

The datasets generated during and/or analyzed during the current study are available via the Sage Bionetworks Synapse portal, syn68716168, with code available from the corresponding author on reasonable request.

## Supporting information



Supporting Information

Supporting Information

Supporting Information
