# Supplementary material for: PAK1 inhibitor NVS‐PAK1‐1 preserves dendritic spines in amyloid/tau exposed neurons and 5xFAD mice
Source: Alzheimers Dement. 2025 Dec 26;21(12):e71033. doi: 10.1002/alz.71033 (PMC12741935; doi:10.1002/alz.71033)
Supplement: Supplementary file 1 — Supporting Information [file ALZ-21-e71033-s003.pdf]

PAK1 inhibitor NVS-PAK1-1 preserves dendritic spines in amyloid/tau exposed neurons and 5xFAD mice

Supplementary methods and results

Figure S1. ^{1}H NMR spectrum of NVS-PAK1-1 in *d$_6$*-DMSO. Spectrum collected in *d$_6$*-DMSO and matched to that previously reported for NVS-PAK1-1 [20].

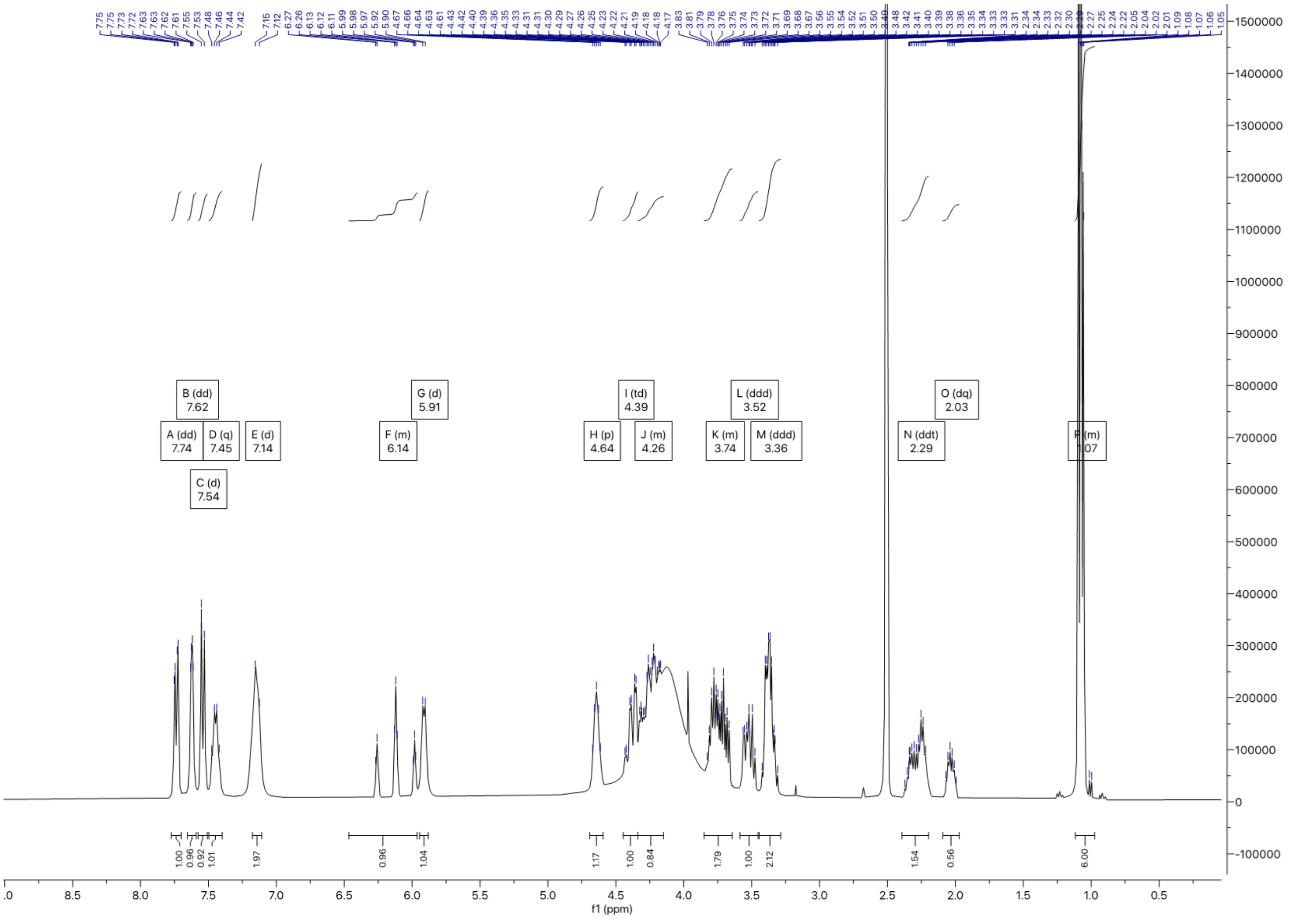

Figure S2. ^{1}H NMR spectrum of NVS-PAK1-1 in MeOD. Identity and purity of NVS-PAK1-1 confirmed.

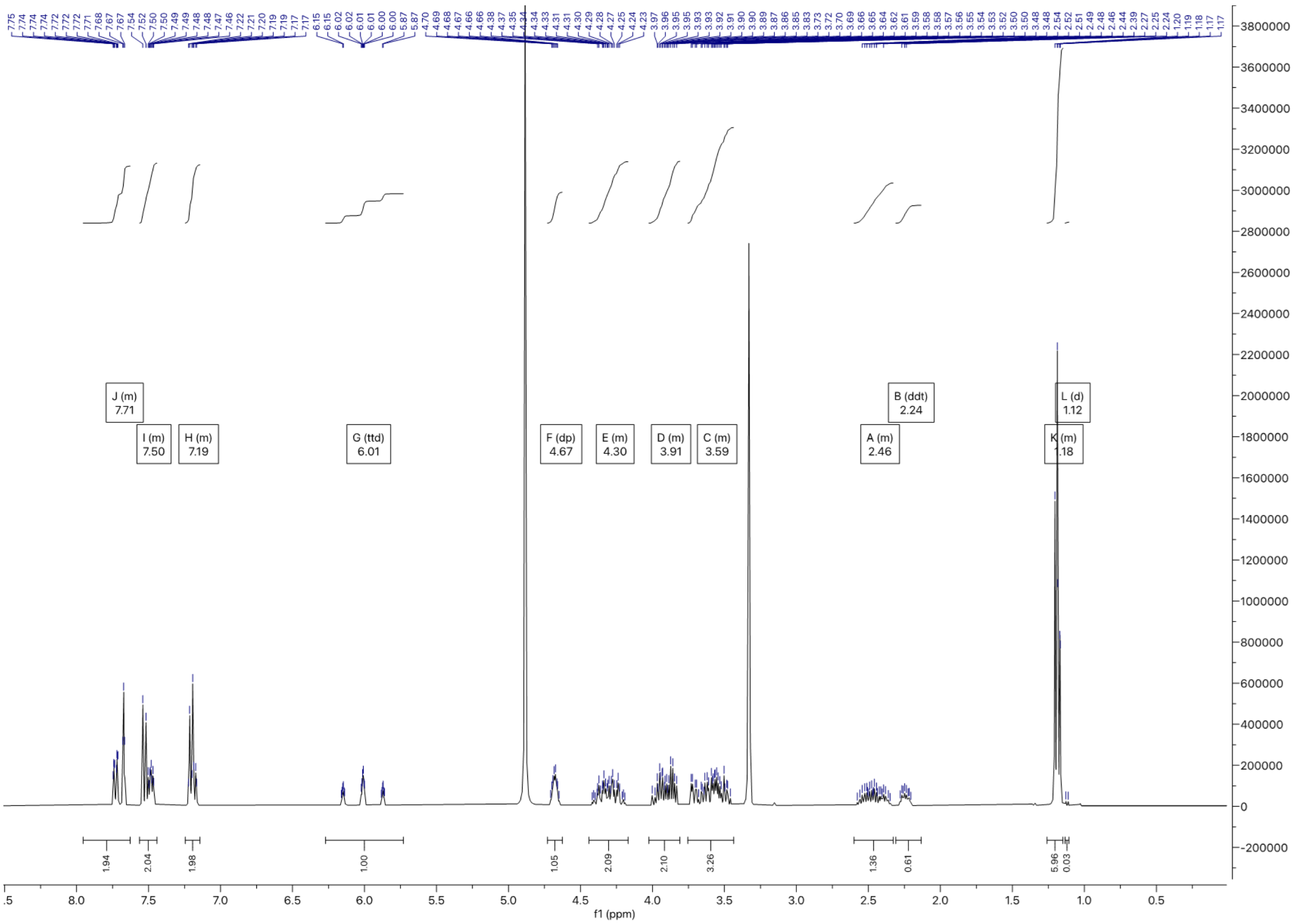

Figure S3. ^{19}F NMR spectrum of NVS-PAK1-1 in MeOD. Fluorine content of NVS-PAK1-1 confirmed.

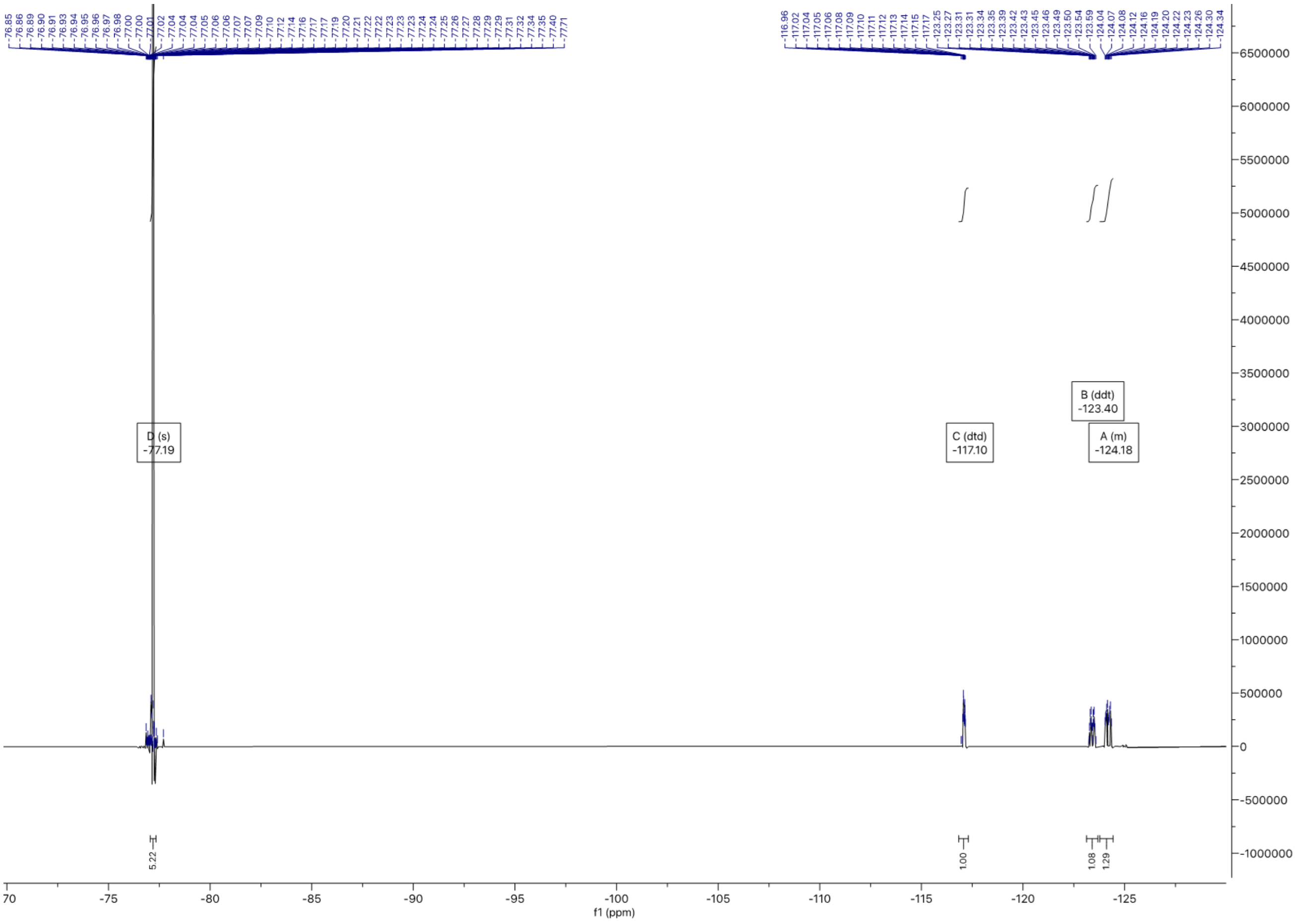

Figure S4. LCMS trace of NVS-PAK1-1. Purity summary for NVS-PAK1-1.

LCMS Sample Purity - Detailed Report

Agilent

**Result set name:**	SingleSample		
**Data file:**	ZDG-AA-16-1.dx	**Project name:**	SGC-UNC
**Sample name:**	ZDG-AA-16-1	**Operator:**	SYSTEM (SYSTEM)
**Instrument:**	LCMS G6160A	**Acquired on:**	2025-03-28 15:10:08-04:00
**Inj. volume:**	1.000 µL	**Location:**	P2-D1
**Acq. method:**	Standard Method_SGC.amx		
**Processing method:**	LC_MS Sample Purity_SGC.pmx		

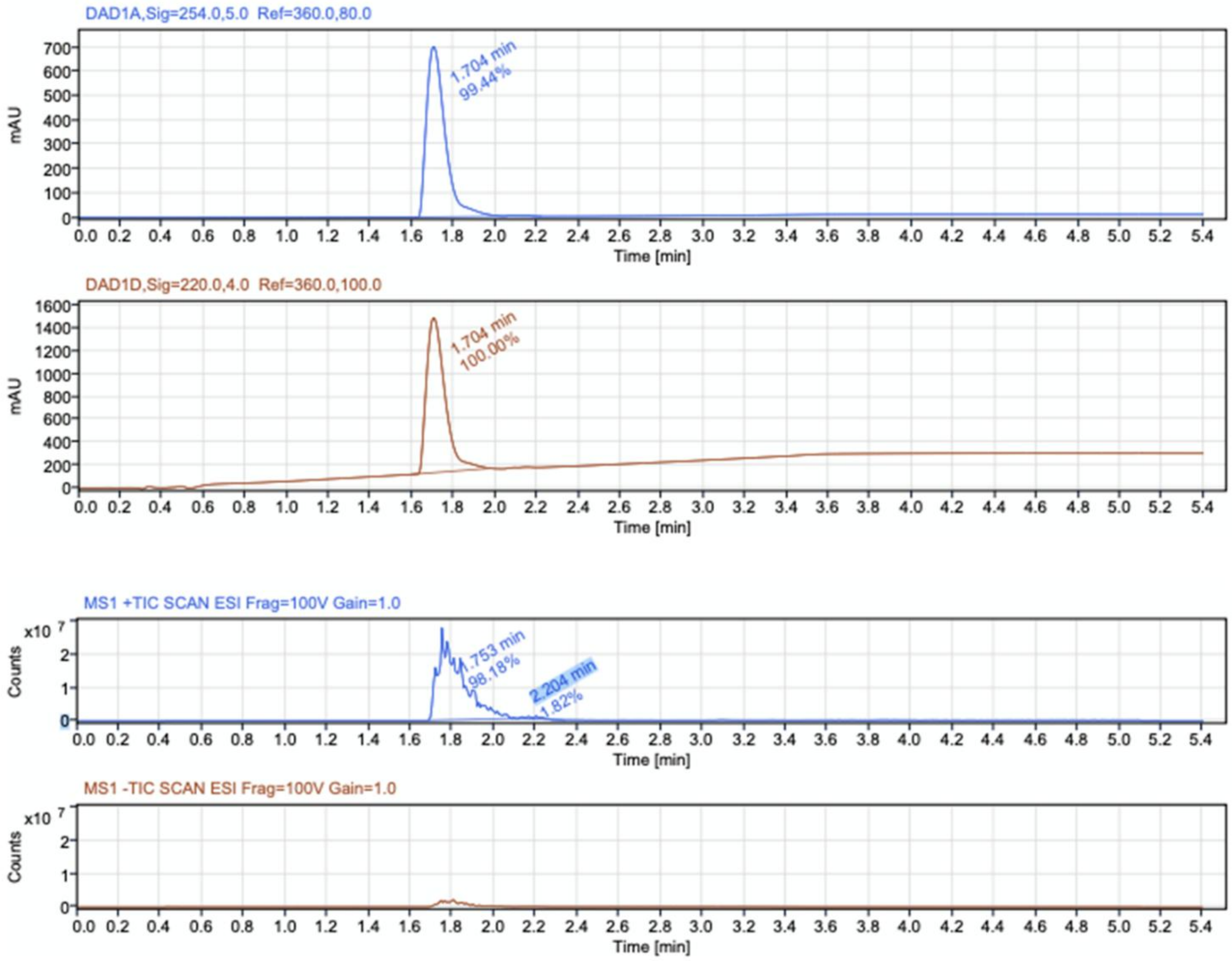

Figure S5. LCMS readout of NVS-PAK1-1. Detailed purity report for NVS-PAK1-1.

LCMS Sample Purity - Detailed Report

Agilent

Peak Results (Area Percent at least 1%)

Signal Name DAD1A

RT (min)	Signal description	Area	Area%	Peak Resolution USP
1.704	DAD1A,Sig=254.0,5.0 Ref=360.0,80.0	4349.4	99.44	
2.088	DAD1A,Sig=254.0,5.0 Ref=360.0,80.0	24.4	0.56	

Signal Name DAD1D

RT (min)	Signal description	Area	Area%	Peak Resolution USP
1.704	DAD1D,Sig=220.0,4.0 Ref=360.0,100.0	8392.7	100.00	

Signal Name MS1 +TIC SCAN ESI Frag=100V Gain=1.0

RT (min)	Signal description	Area	Area%	Peak Resolution USP
1.753	MS1 +TIC SCAN ESI Frag=100V Gain=1.0	209624799.4	98.18	
2.204	MS1 +TIC SCAN ESI Frag=100V Gain=1.0	3887104.4	1.82	

Signal Name MS1 -TIC SCAN ESI Frag=100V Gain=1.0

RT (min)	Signal description	Area	Area%	Peak Resolution USP
	MS1 -TIC SCAN ESI Frag=100V Gain=1.0			

Signal Description: MS1 +TIC SCAN ESI Frag=100V Gain=1.0

Peak RT: 1.753 min **Area %:** 98.18%

Peak Details (Area Percent at least 1%)

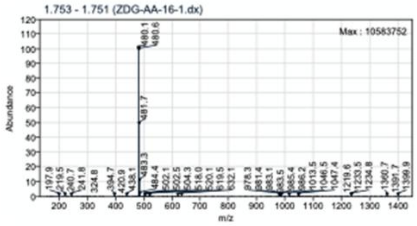

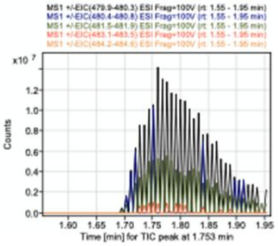

Peak RT: 2.204 min **Area %:** 1.82%

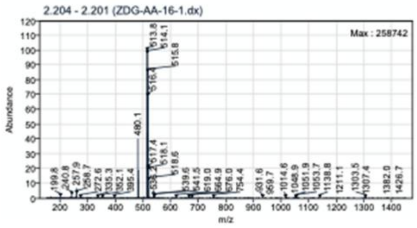

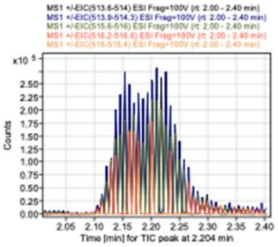

Signal Description: DAD1A,Sig=254.0,5.0 Ref=360.0,80.0

Peak RT: 1.704 min **Area %:** 99.44%

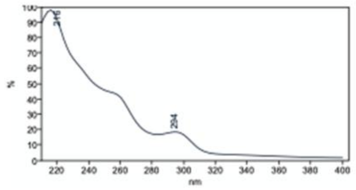

Figure S6. Chronic oral administration of NVS-PAK1-1 in 5xFAD mice shows group differences expression of proteins and overlapping differential expression patterns.

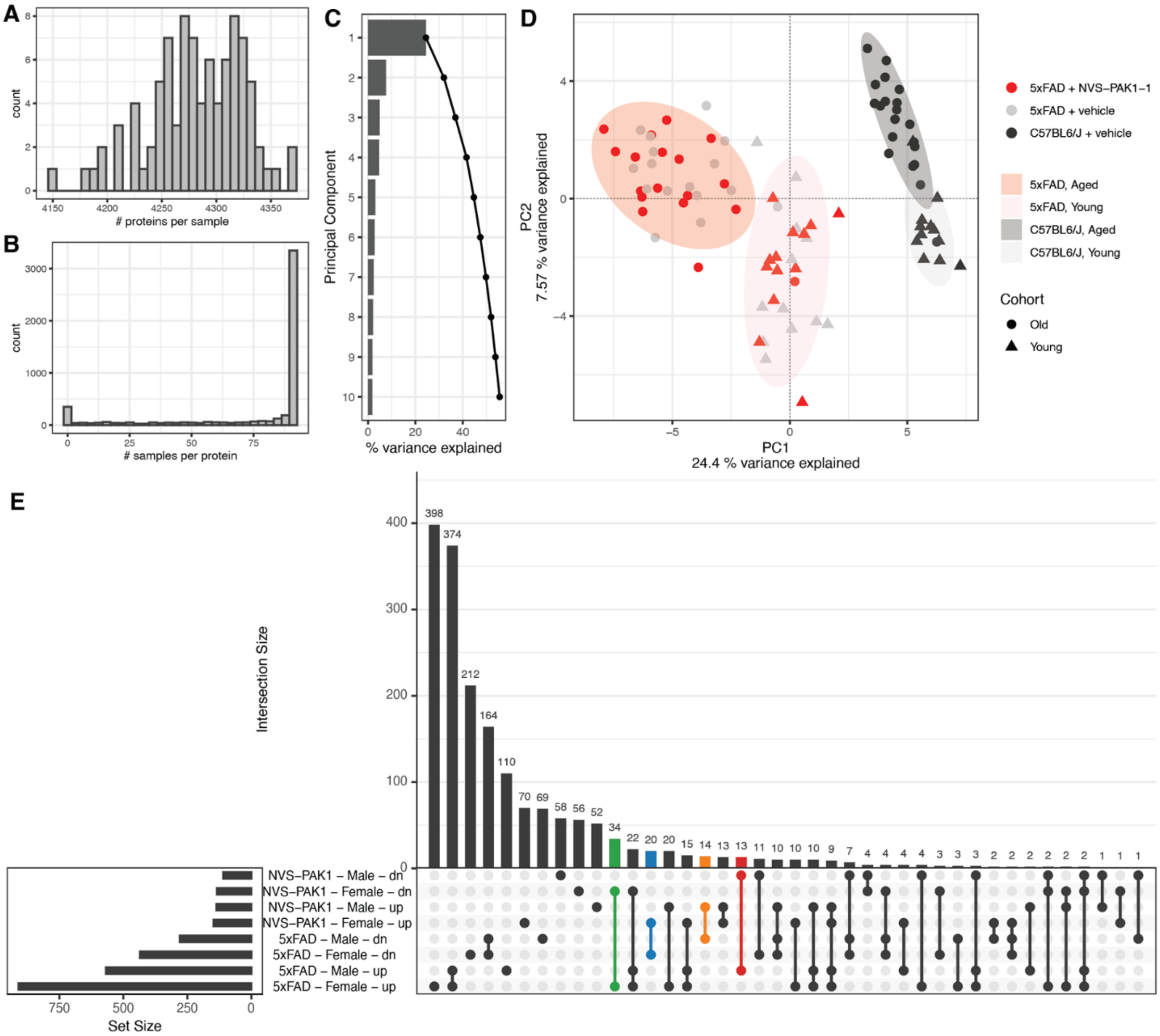

(**A-E**) Treatment with twice daily oral administration of NVS-PAK1-1 (100 mg/kg or vehicle control, BID, PO) was initiated in young (2-3 month aged) or aged (9 month aged) female and male 5xFAD mice and compared to vehicle-treated C57BL6/J (WT) littermate controls and 5xFAD vehicle controls. Terminal brain and tissue samples were collected at 4 h post dose on the final day of treatment which was at 6 months of age (young) or 10 months of age (aged; 30-day treatment). **(A)** The distribution of number of proteins per sample. **(B)** The distribution of

sample number with a given protein. **(C)** Principal component eigenvalues for the first 10 components. **(D)** Scatterplot of principal components 1 and 2. Individual mice are colored by treatment, either NVS-PAK1-1 treated 5xFAD mice (red), vehicle treated 5xFAD mice (gray), or vehicle treated C57BL6/J mice (black). Shapes define the young (circle) or aged (triangle) cohort mice. Ellipses represent a 95% CI around the centroid for each of four groups: 5xFAD aged (peach), 5xFAD young (light peach), C57BL6/J aged (gray), C57BL6/J young (light gray). **(E)** Upset plot of overlaps of differential expression for two comparisons: vehicle treated 5xFAD vs vehicle treated C57BL6/J (**5xFAD**) and NVS-PAK1-1 treated 5xFAD vs vehicle treated 5xFAD **(NVS-PAK1-1)** in the union of DE proteins in the young and aged cohorts. Differential comparisons conducted separately on male and female groups and are split into upregulated and downregulated proteins. Colored overlaps (green, orange, blue, red) are within the same gender, where the genes in each comparison go in the opposite direction, i.e. up in 5xFAD and down in NVS-PAK1-1.

Figure S7. Chronic oral administration of NVS-PAK1-1 in aged female and male 5xFAD mice does not show PAK1 inhibition.

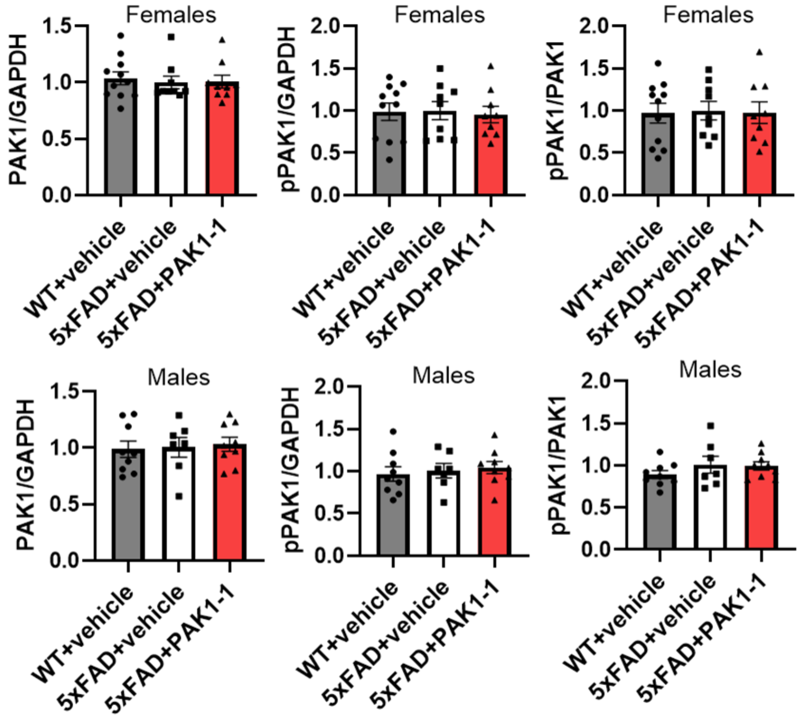

Treatment with twice daily oral administration of NVS-PAK1-1 (100 mg/kg or vehicle control, BID, PO) was initiated in aged (9 month aged) female **(upper)** and male **(lower)** 5xFAD mice and compared to vehicle-treated WT littermate controls and 5xFAD vehicle controls. Terminal brain and tissue samples were collected at 4 h post dose on the final day of treatment which was at 10 months of age (31-day treatment). Brains were collected and processed for PAK1 activity assessment. PAK1 activity as measured by total pPAK1 expression and by the pPAK1/PAK1 ratio relative to vehicle-treated age- and sex-matched 5xFAD controls. The relative expression of PAK1 and pPAK1 were normalized within internal control GAPDH. The fold changes of PAK1, pPAK1, and pPAK1/PAK1 were compared with sex-matched 5xFAD vehicle treated controls and analyzed by one-way ANOVA.

Figure S8. Chronic oral administration of NVS-PAK1-1 in 5xFAD mice shows TREAT-AD biological domain differences across the different cohorts.

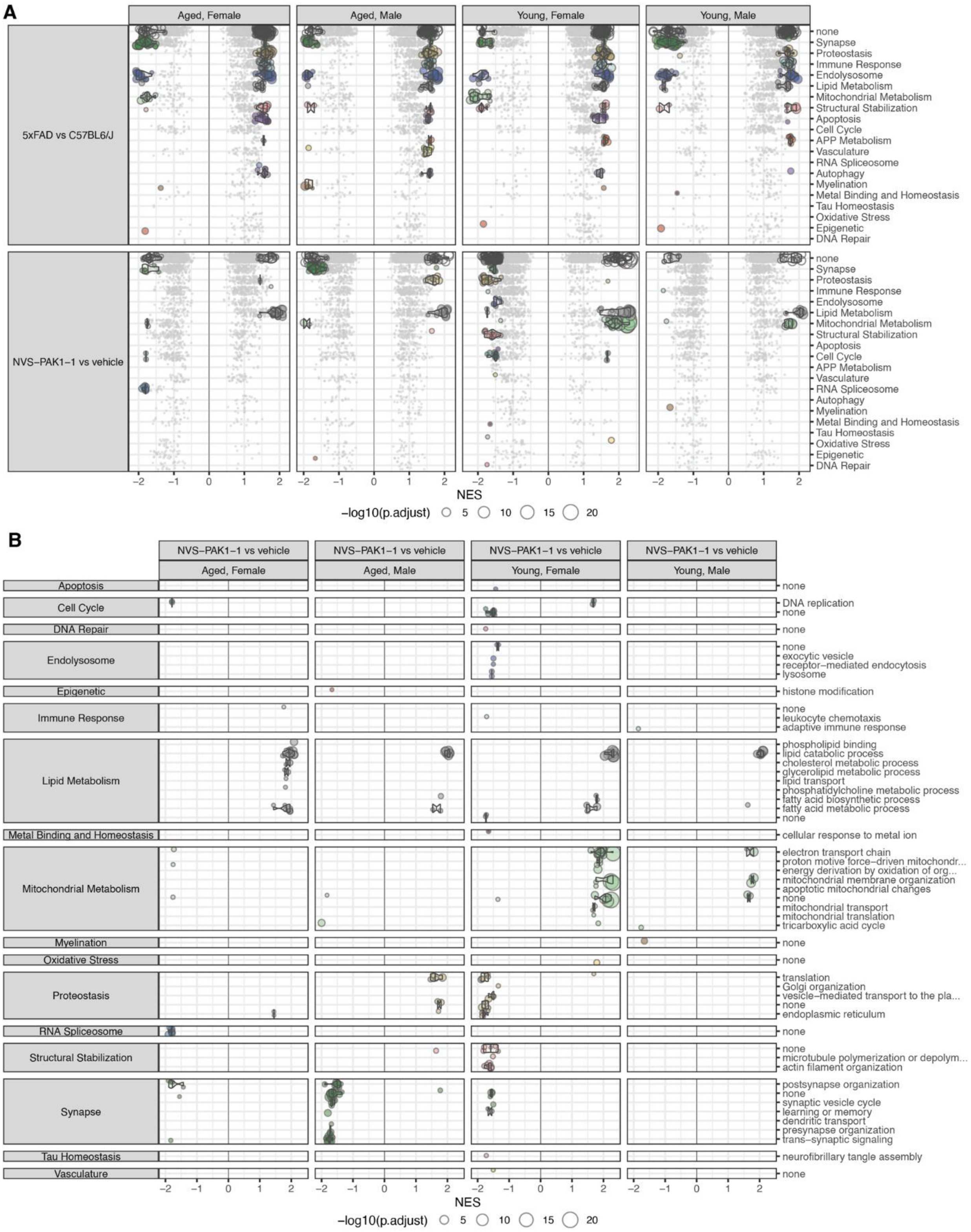

(A, B) Treatment with twice daily oral administration of NVS-PAK1-1 (100 mg/kg or vehicle control, BID, PO) was initiated in young (2-3 month aged) or aged (9 month aged) female and male 5xFAD mice and compared to vehicle-treated C57BL6/J (WT) littermate controls and 5xFAD vehicle controls. Terminal brain and tissue samples were collected at 4 h post dose on the final day of treatment which was at 6 months of age (young) or 10 months of age (aged; 30-day treatment). **(A)** Gene set enrichment analysis (GSEA) of differentially expressed proteins in the four cohorts for two comparisons: vehicle treated 5xFAD vs vehicle treated C57BL6/J (5xFAD vs C57BL6/J) and NVS-PAK1-1 treated 5xFAD vs vehicle treated 5xFAD (NVS-PAK1-1 vs vehicle). The logFC-signed negative log10 adjusted p-value for each protein was used to rank proteins for GSEA. Gene Ontology (GO) terms that were significantly enriched by either GSEA or overrepresentation analysis were mapped onto the TREAT-AD biological domains of AD that map existing ontologies onto the AD endophenotypic space. Significant (padj < 0.05) terms are colored by biological domain, and circle size represents the enrichment significance ($-\log_{10}$padj). **(B)** Gene set enrichment analysis (GSEA) of differentially expressed proteins in the NVS-PAK1-1 vs vehicle comparison, with significant GO terms mapped onto the TREAT-AD biological subdomains within the overarching domains. Significant (padj < 0.05) terms are colored by biological domain, and circle size represents the enrichment significance ($-\log_{10}$padj).

Figure S9. Chronic oral administration of NVS-PAK1-1 does not alter plasma concentrations of Aβ40, Aβ42, Nfl, or soluble or insoluble brain Aβ40 or Aβ42 in aged 5xFAD mice.

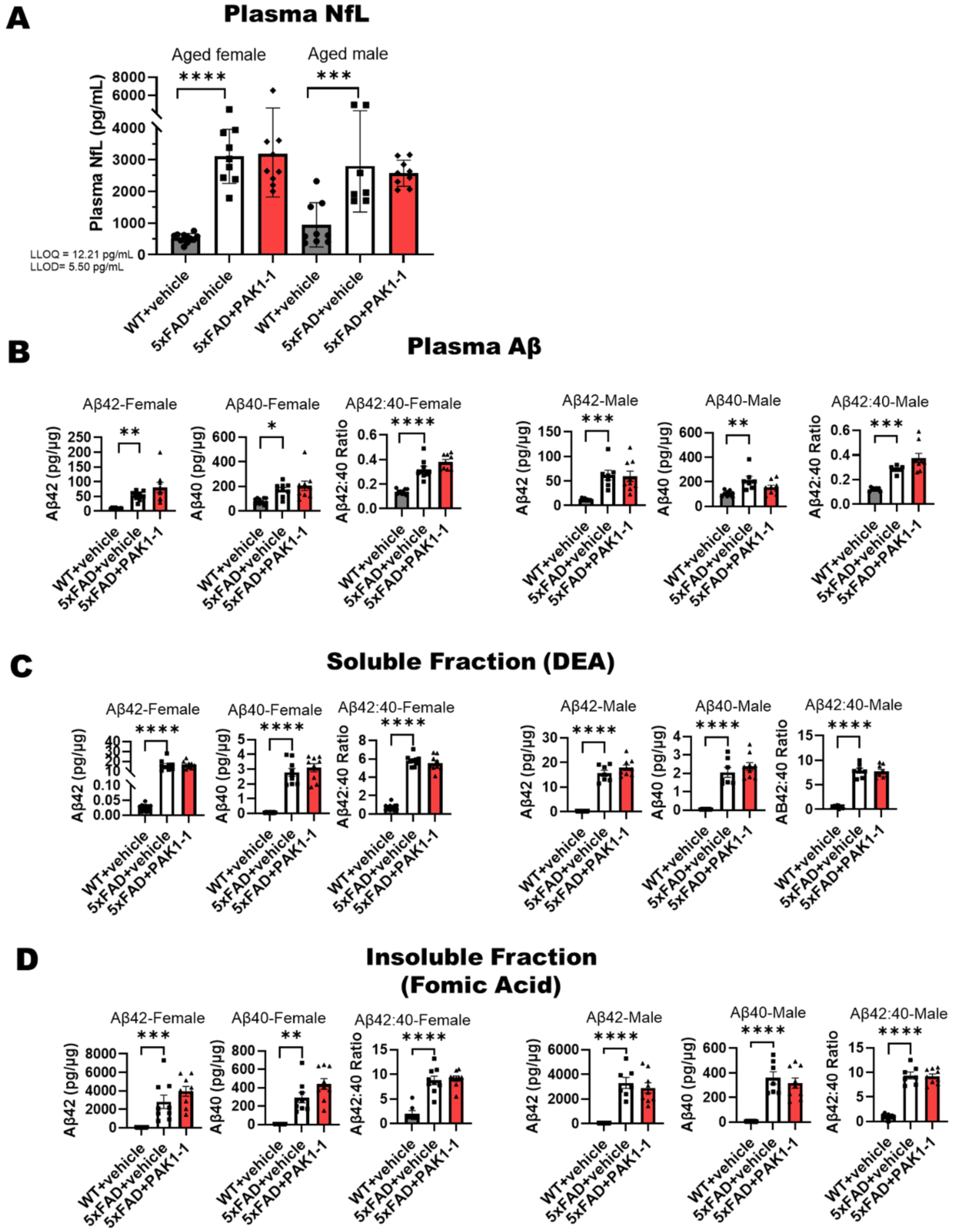

(A-D) Treatment with twice daily oral administration of NVS-PAK1-1 (100 mg/kg or vehicle control, BID, PO) was initiated in aged (9 month aged) female and male 5xFAD mice and compared to vehicle-treated WT littermate controls and 5xFAD vehicle controls. Terminal brain and tissue samples were collected at 4 h post dose on the final day of treatment which was at 10 months of age (31-day treatment). (**A)** As expected, there was an increase of plasma NfL in 5xFAD mice relative to age- and sex-matched vehicle-treated WT littermate controls. There was no significant difference in NVS-PAK1-1 treated 5xFAD mice relative to vehicle-treated 5xFAD. (**B-D)** Plasma Aβ40 and Aβ42 (**B**), soluble fraction (**C**) and insoluble fraction brain (**D**). Aβ40 and Aβ42 were quantified by MSD ELISA are illustrated for young females (**left**) and young males (**right**). Vehicle-treated 5xFAD mice demonstrated the expected increases in Aβ 40, Aβ 42, and Aβ42/40 relative to vehicle-treated age- and sex-matched WT littermate controls. There was no significant difference in NVS-PAK-1 treatment in 5xFAD mice relative to age- and sex-matched 5xFAD vehicle-treated controls. Data are presented as mean±st.dev (n=4-8 per sex per age per treatment). * $p < 0.05$, ** $p < 0.01$, *** p < 0.00,1 by one-way ANOVA with Šídák's multiple comparisons test.
